# Full-Signal Ultrahigh-Resolution NMR by Parameter Estimation

**DOI:** 10.1021/acs.analchem.5c03446

**Published:** 2025-11-08

**Authors:** Simon G. Hulse, Mathias Nilsson, Gareth A. Morris, Mohammadali Foroozandeh

**Affiliations:** † Chemistry Research Laboratory, 6396 University of Oxford, 12 Mansfield Rd, Oxford OX1 3TA, U.K.; ‡ Department of Chemistry, 5292 University of Manchester, Oxford Road, Manchester M13 9PL, U.K.

## Abstract

Pure shift NMR spectra, in which multiplet structure is suppressed, are widely used but exact a high price in sensitivity. Here we present CUPID (Computer-assisted Undiminished-sensitivity Protocol for Ideal Decoupling), which uses parametric estimation to produce pure shift NMR spectra from easily acquired 2D J-resolved (2DJ) data sets. Unlike previous practical methods for broadband pure shift NMR, it makes use of all of the available signal. CUPID is therefore effective even at sample concentrations where current methods are too insensitive to yield usable spectra. As an additional benefit, the estimation method used allows the extraction of individual multiplet structures from overlapping spectra. CUPID is freely available through NMR Estimation in Python (NMR-EsPy), an open-source package with a simple-to-use API, and comes with a graphical user interface that is accessible via Topspin, a widely used NMR software platform.

## Graphical abstract

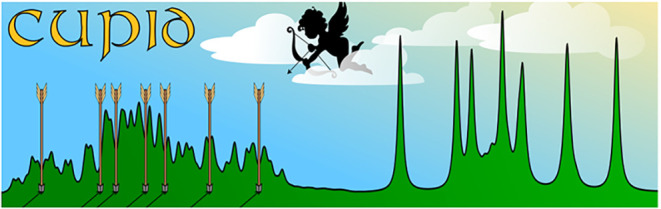

## Introduction

Proton NMR spectroscopy has a strong claim to be the single most important analytical methodology in synthetic chemistry. It is quick, simple, and sensitive, and most chemists are expert at extracting chemical information from the spectra obtained. Its primary weakness is its limited resolution: the low electron density around the hydrogen atom makes for a very narrow range of chemical shifts, and the ubiquitous coupling interactions between protons typically lead to multiplet structures that are many times wider than the natural peak width. If this multiplet structure is suppressed, the resolution of proton NMR can be increased by up to an order of magnitude. 2D J-resolved (2DJ) spectroscopy^1,2^ is a well-known technique which can in principle be employed to produce such broadband homodecoupled (“pure shift”) NMR spectra. This is achieved by applying a 45° “tilt” (formally a shear) to the 2D J-spectrum, separating the effects of chemical shifts and scalar couplings orthogonally along the direct and indirect axes, respectively. A projection onto the direct dimension then yields what ought to be a pure shift spectrum. Unfortunately, the 2DJ pulse sequence (a simple spin echo) produces signals which are phase-modulated with respect to both time dimensions, which makes it impossible to generate a spectrum with desirable 2D absorption-mode lineshapes via the 45°-tilt approach.^3^ Indeed, unless special processing is used, the 45° projection is zero, as the positive and negative contributions in the natural “phase twist” peak shapes perfectly cancel each other. To overcome this, 2DJ spectra are conventionally presented in magnitude (absolute-value) mode. Both the 2D spectrum and the resulting 45° pure shift projection feature broad peaks with gross distortions, sacrificing almost all of the desired gain in resolution. Manipulating the time-domain data using methods like pseudo-echo reshaping^4^ and sine-bell apodization^5^ can help to suppress the dispersive contributions to the peaks, but only at a high cost in sensitivity and in distortion of relative peak integrals. Here we revisit this problem, and show that by parametric estimation it is possible to construct an undistorted absorption-mode pure shift spectrum from experimental 2DJ data without any of the signal sacrifice that previous methods have required.

Most practical pure shift experiments involve the construction of an interferogram from the initial segments of FIDs measured after the application of a J-refocusing element in a 2D-mode experiment.^6^ Prominent examples of J-refocusing elements include that of Zangger and Sterk;^7,8^ Bilinear Rotational Decoupling (BIRD);^9,10^ and Pure Shift Yielded by Chirp Excitation (PSYCHE).^11,12^ In an alternative approach, variants of the classic 2DJ experiment that incorporate J-refocusing elements have been used to form data sets which consist of P- and N-type hypercomplex pairs,^3^ yielding 2DJ spectra with absorption-mode lineshapes.^13,14^ The key drawback of using J-refocusing elements is that most of the available signal has to be discarded, since only a small subset of the spins present actively contribute to the detected signal. AI methods have been used^15,16^ to reduce slightly the amount of experimental data needed to construct a pure shift spectrum by the PSYCHE method, but they only recover a small proportion of the sensitivity loss incurred by the use of the PSYCHE J-refocusing element.

In principle, all of the information needed to construct a pure shift spectrum is present in an experimental 2DJ data set; the challenge is to extract it in interpretable form. One way to do this is to estimate the parameters (frequency, intensity, phase, line width) that describe all of the signals in the 2DJ data set, allowing a pure shift spectrum with absorption-mode lineshapes to be calculated. The great advantages of such an approach are that the resultant spectrum is fully quantitative, and that all of the available nuclear signal contributes to the spectrum, rather than just a fraction of it, as in existing broadband pure shift methods. This concept has been demonstrated in previous techniques such as ALPESTRE,^17,18^ a similar procedure from Mutzenhardt et al.,^19^ and the Filter Diagonalization Method (FDM),^20,21^ though they have not yet found widespread use. In such methods, each individual time slice, either in the direct or indirect dimension, is estimated and subsequently back-propagated into negative time to yield a full echo. Fourier Transformation (FT) of the full echo produces a 2DJ spectrum with pure 2D absorption lineshapes, such that processing by the 45°-tilt approach yields a phase-sensitive pure shift spectrum with absorption-mode 1D lineshapes.

This work introduces a Computer-assisted Undiminished-sensitivity Protocol for Ideal Decoupling (CUPID), which is accessible as part of NMR Estimation in Python (NMR-EsPy),^22^ an open source package which has been presented previously in the context of 1D NMR data estimation.^23^ In contrast to previous methods, the procedure used considers the 2DJ data set holistically, rather than slice by slice, reducing the impact of noise on the accuracy of parameter estimation. Additional benefits include the ability to extract multiplet structures, and to edit out solvent signals and strong coupling artifacts.

## Theory

A 2DJ data set can be modeled as a summation of *M* signals, each being an exponentially damped complex sinusoid defined by six parameters: amplitude *a*, phase ϕ, two angular frequencies (ω_1_, ω_2_), and two damping factors (η_1_, η_2_)­

1 $$y_{n_{1},n_{2}}=\sum_{m=1}^{M}a_{m}e^{i\phi_{m}}\prod_{d=1}^{2}e^{\left(i\omega_{d,m}-\eta_{d,m}\right)\left(n_{d}-1\right)\tau_{d}}+w_{n_{1},n_{2}}$$

∀*n*_1(2)_ ∈ {1, 2, ..., *N*_1(2)_}, with *N*_1(2)_ being the number of data points in the indirect (direct) dimension and τ_1(2)_ being the dwell time in the relevant dimension. *w*_*n*_1_,*n*_2__ accounts for contamination of the FID by noise. The estimation procedure used as part of CUPID is similar to the 1D approach presented before;^23^ it comprises three parts:

1. Determination of an estimate of the model order *M*. If possible, this is done by computing the minimum description length (MDL) criterion of the first direct-dimension FID in the data set.^24,25^ In cases where the direct-dimension spectrum is particularly crowded, an appropriate value should be provided manually by the user.
2. Generation of an initial guess of parameters, using the modified matrix enhancement and matrix pencil method (MMEMPM).^26,27^

3. Subjection of the initial guess to numerical optimization, with the cost function consisting of the residual sum-of-squares between the model and data, regularized by the variance of the model signal phases.

It is usually helpful to generate frequency-filtered “sub-FIDs” in the direct dimension, as the computational demands scale unfavorably with the number of data points and the model order. A similar segmentation of frequency space is used, for the same reasons, in the FDM.^28^ The regions of interest from which the sub-FIDs are produced are currently specified manually by the user, but this could easily be automated.

All first-order signals (those which are expected under the weak coupling approximation) in 2DJ data sets have direct- and indirect-dimension frequencies which are intimately linked; they can be expressed as ω_1_ = ω_D_ and ω_2_ = ω_C_ + ω_D_, where ω_C_ is the central frequency of the multiplet that the signal belongs to (i.e., the relevant spin’s resonance frequency, its chemical shift), and ω_D_ is a displacement from ω_C_, caused by scalar couplings with other spins. The resonance frequencies ω_C_ of the spins are therefore equal to ω_2_ – ω_1_. This is what the 45° tilt achieves: frequencies in the direct dimension are transformed from ω_2_ to ω_2_ – ω_1_.

Given an estimate of the parameters that describe a 2DJ data set, one can construct a synthetic “–45° signal” ***ỹ*** which is the inverse Fourier transform of the pure shift spectrum

2 $${ỹ}_{n}=\sum_{m=1}^{M}{â}_{m}e^{i{\mathrm{\phi ̂}}_{m}}e^{\left(i\left({\mathrm{\omega ̂}}_{2,m}-{\mathrm{\omega ̂}}_{1,m}\right)-{\mathrm{\eta ̂}}_{2,m}\right)\left(n-1\right)\tau_{2}}$$

with *n* ∈ {1, ..., *N*_2_}. The ^ symbol is used to emphasize quantities which have been estimated. Conventional FT-based processing of the −45° signal produces a quantitative pure shift spectrum with absorption-mode lineshapes and the same peak integrals as a pulse-acquire spectrum measured for the same sample under equivalent experimental conditions. Spectra produced by CUPID do not feature any of the noise found in experimental spectra. However, it is of course incorrect to think of these spectra as “noiseless”; the noise in the input data is translated into small uncertainties in the amplitudes, frequencies etc. of the pure shift peaks, being incorporated in the errors associated with the estimated parameters. Further errors can arise when a model with fewer signals than the true number is used (i.e., when under-fitting), and when additional spurious signals are incorporated into the model, which do not correspond to genuine signals in the data.

Because holistic estimation of the 2DJ datset provides access to the signal frequencies in both dimensions, CUPID is able to extract individual multiplet structures: any pair of signals *i*, *j* ∈ {1, ..., *M*} can be assumed to be part of the same multiplet if

3 $$\left|\left({\mathrm{\omega ̂}}_{2,i}-{\mathrm{\omega ̂}}_{1,i}\right)-\left({\mathrm{\omega ̂}}_{2,j}-{\mathrm{\omega ̂}}_{1,j}\right)\right|<\epsilon$$

where ϵ is a threshold to account for uncertainty in the estimation. A lower bound for ϵ is the digital resolution in the less well resolved of the two dimensions. i.e.

4 $$\epsilon =\max_{d\in \left\{1,2\right\}}\left(1/\tau_{d}N_{d}\right)$$

In practice, ϵ sometimes needs to be increased manually to resolve multiplets effectively. Multiplet extraction also allows for the identification of certain types of unwanted signal, which can be discarded from the model prior to construction of the pure shift spectrum. Any estimated signal which satisfies both of the following “non-first-order criteria”:

1. it is not grouped with any other signal by multiplet identification,
2. its indirect-dimension frequency is sufficiently far from zero (|ω̂_1_| > ϵ),

is likely to arise from fitting either a strong coupling artifact or a noise component.

## Experimental Section

Two examples of the application of CUPID are provided here; the data sets featured were acquired from (a) 70 mM quinine (Figure 1a) in CD_3_OD; and (b) low-concentration (2 mM) 17β-estradiol (Figure 1b) in DMSO-*d*_6_. Both data sets were acquired at 298 K, using 500 MHz Bruker spectrometers. For the quinine data, an Avance II+ 500 spectrometer equipped with a 5 mm BBO probe, operating at 500.13 MHz, was used, while an AV III HD 500 spectrometer equipped with a 5 mm TBO probe, operating at 500.13 MHz, was used for the estradiol data. The 2DJ experiments were performed using the jresqf pulse sequence, part of Bruker’s standard library. Both data sets were acquired as 128 × 8k complex grids, with estimated acquisition times being 38 and 28 min, respectively. An additional 2DJ data set was acquired on a higher-concentration estradiol sample (10 mM), to ensure that an intelligible contour plot of the 2DJ spectrum could be produced (*vide infra*). An accompanying PSYCHE experiment was also performed on the 2 mM estradiol sample for comparison. Parameters equivalent to the 2DJ experiment were used where applicable; further experimental details are provided in the Supporting Information (SI).

**Figure 1:**
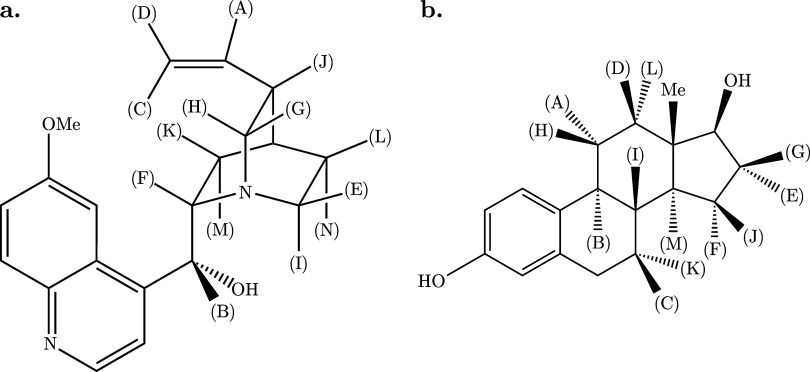
Structures of (a) quinine and (b) 17β-estradiol. Proton environments giving rise to the signals shown in Figures 2 and 3 are labeled with bracketed upper-case letters.

The NMR-EsPy package was used to generate the results presented in this work.^22^ The package can be utilized either through Python scripts, or by running the accompanying graphical user interface. For details on usage, the reader is directed to the documentation: https://foroozandehgroup.github.io/NMR-EsPy/. The procedure for acquiring each result involved the following steps:

1. The 2DJ data set was processed, by applying frequency-domain phase correction to each direct dimension FID. This was achieved by determining appropriate zero- and first-order phases to correct the first slice, and then applying the same correction to the rest.
2. Spectral regions in the direct dimension were specified within which peaks reside, and a filtered sub-FID was generated for each region.
3. Each sub-FID was estimated in turn, using the steps outlined above.
4. Multiplet structures were identified; in cases where the default threshold ϵ (eq 4) produced undesirable groupings, a larger threshold was provided manually. Signals satisfying both of the nonfirst-order criteria were removed from the result.
5. A pure shift spectrum was produced using eq 2, along with spectra of the individual multiplet structures.

## Results

The outcome of applying CUPID to the nonaromatic signals of the quinine data set is presented in Figure 2. For comparison, a spectrum generated by applying the classical magnitude mode 45°-tilt approach to the same 2DJ data set is also shown. CUPID was able to produce a clean pure shift spectrum, with one distinct peak for each proton environment, with far greater resolution than the 45°-tilt spectrum. Because the time-domain weighting required for magnitude processing sacrifices a lot of signal, especially for signals with short *T*_2_, the absolute amplitudes of the signals in the 45° projection are reduced by over an order of magnitude, necessitating scaling up by a factor of 30 in Figure 2a. The peak widths are much greater in Figure 2a because of the need to apply severe symmetrizing weighting functions, in this case a sine bell, when using magnitude display. Furthermore, as strong coupling responses are dropped in the parameter estimation process, artifact peaks such as that labeled with an asterisk in panel a do not appear in the CUPID pure shift spectrum. Multiplet extraction was performed using the default threshold of ϵ = 1/(τ_2_ *N*_2_) ≈ 0.92 Hz. Through this, it was possible to distinguish between the signal due to residual water and the multiplet structure of proton (D), for which a difference in center frequencies of 1.27 Hz was determined. Of course, significant overlap between (D) and HDO still features in the pure shift spectrum generated by CUPID (see panel b). (It is possible to produce a clean peak for (D) by editing out the contribution from HDO, as illustrated in the SI, but such manipulations should not be undertaken lightly and should always be clearly documented).

**Figure 2:**
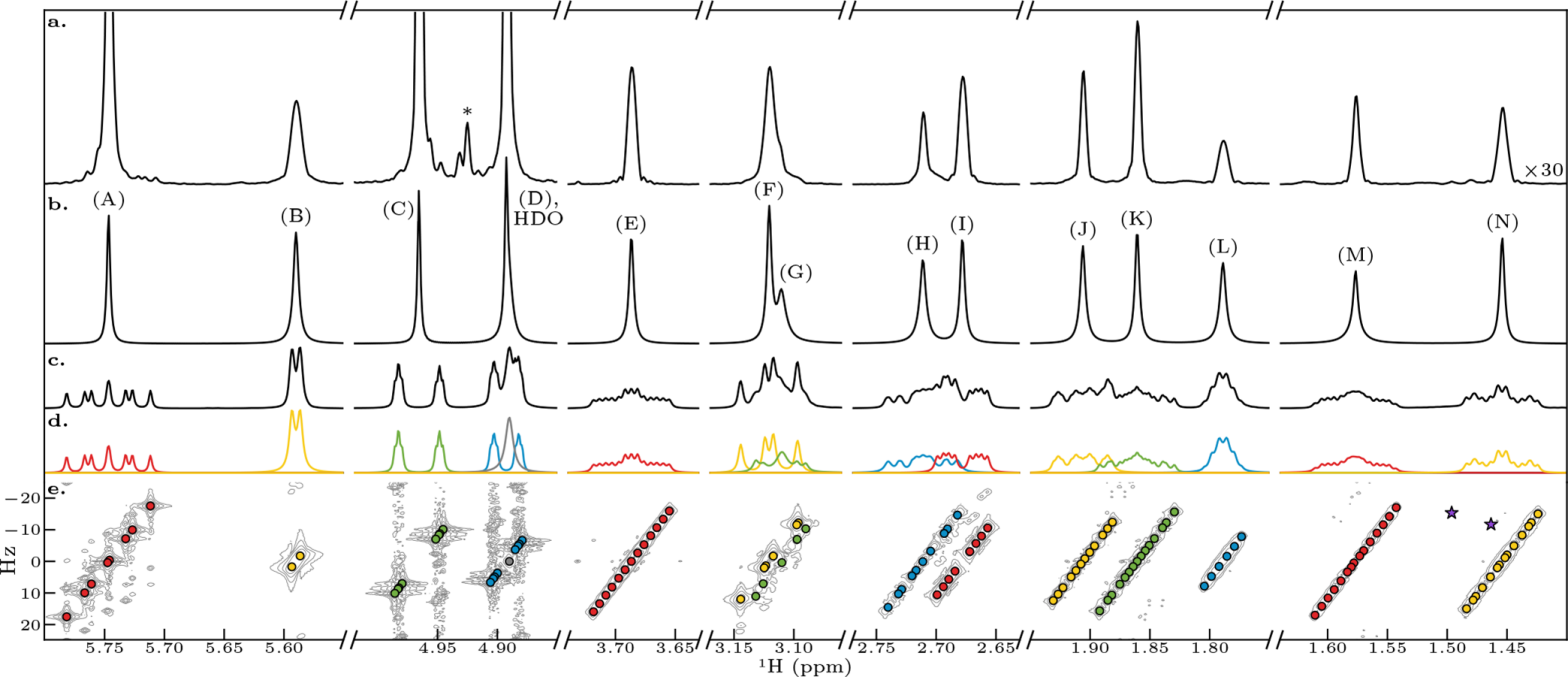
Application of CUPID to the nonaromatic regions of a 2DJ data set for a 70 mM sample of quinine in CD_3_OD. (a) Pure shift spectrum generated using the classical magnitude-mode tilt-and-project approach. An example of an artifact that results from strong coupling effects is marked with an asterisk. (b) Pure shift spectrum constructed using CUPID. (c) FT of the first direct-dimension FID of the 2DJ datset. (d) Multiplets extracted using a threshold of 0.92 Hz, equal to the digital resolution of the direct dimension. The estimated signal corresponding to water is colored gray. The 1D spectra in panels (b–d) were all subjected to 0.29 Hz exponential line-broadening. (e) Magnitude-mode 2DJ spectrum, with the positions of estimated signals marked by colored points. The positions of signals that existed after estimation, but which were automatically purged based on the first-order criteria are marked as purple stars.

Figure 3 shows how CUPID performed on the dilute estradiol sample. The PSYCHE spectrum (Figure 3a) has a very low signal-to-noise ratio (SNR), reflecting the low sample concentration and the signal sacrificed by the J-refocusing element. No data are shown for methods such as 45° projection of pure absorption 2DJ spectra,^13,14^ because these have substantially lower sensitivity than PSYCHE and hence would produce no usable data here. CUPID, in contrast, successfully generates a pure shift spectrum (Figure 2b) despite the poor SNR and the complex overlapping multiplet structures present. It should be emphasized that CUPID is not a cosmetic procedure: it separates the experimental data into identified peaks, which are used to construct the pure shift spectrum as in Figure 2b, and residual, as in Figure 2e. Comparison between the pure shift spectrum and the residuals allows the spectroscopist to gauge the significance of the peaks seen, just as comparison between peaks and baseline noise is used in a conventional spectrum.

**Figure 3:**
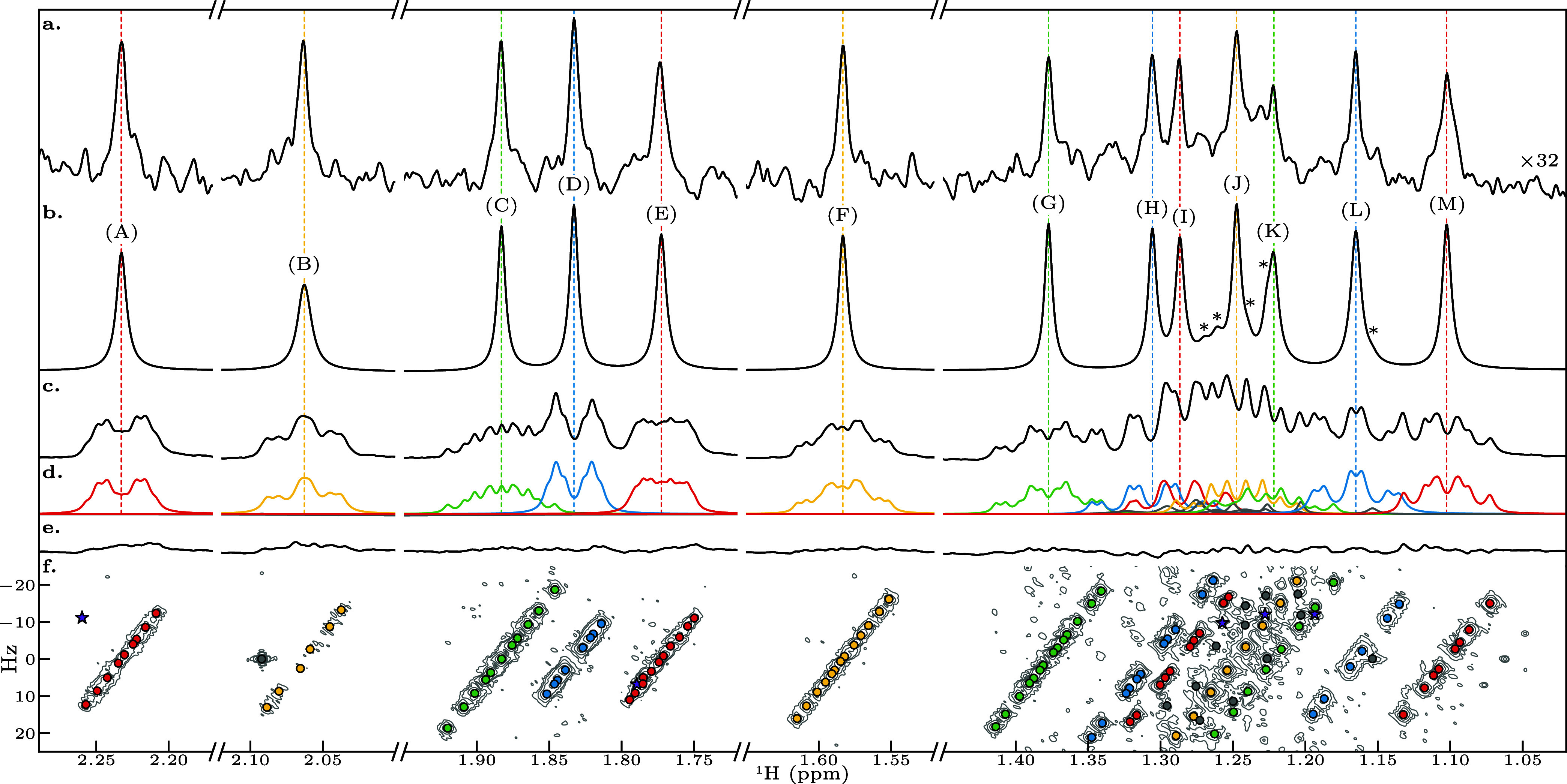
Application of CUPID and PSYCHE to a 2DJ data set for 2 mM 17β-estradiol in DMSO-*d*_6_. (a) PSYCHE spectrum of the sample, scaled up by a factor of 32 so that peak intensitites are comparable to those for CUPID. (b) Pure shift spectrum generated by CUPID. Contributions from estimated signals which correspond to strong coupling artifacts are marked with asterisks. (c) Conventional 1D spectrum, obtained by FT of the first FID of the 2DJ datset. (d) Multiplet structures extracted, using a threshold of 2 Hz. All the 1D spectra in panels a–d were subjected to exponential apodization with a line-broadening factor of 0.78 Hz before zero-filling and FT. (e) Residual of the first increment of the 2DJ data set, obtained by subtracting the sum of all estimated signals from the first 2DJ FID. (f) Magnitude-mode 2DJ spectrum, with the locations of estimated signals marked by colored points. Those which are believed to arise from strong couplings/noise are colored gray, while those which were removed based on the first-order criteria are marked as purple stars. N.B. The spectrum in panel f was acquired using a higher-concentration (10 mM) sample than the data presented in the other panels; after sine-bell apodization, the data from the 2 mM, sample was too noisy for a usable contour plot to be generated.

In the region 1.26 ppm–1.15 ppm, there are estimated signals which do not correspond to first-order estradiol signals; these arise from fitting strong coupling artifacts, and are denoted by gray points in panel f. A number of other estimated signals were removed automatically from the final result as part of the multiplet identification process; their locations are marked by purple stars. Small strong coupling responses that could not be rejected with confidence led to the presence of low-intensity shoulders on the peaks in the pure shift spectrum; these are labeled with asterisks in panel b. The total time to process all the regions considered in the estradiol data set was just under 4 min, using a workstation featuring an Intel Core i9–7920X CPU @ 2.9 GHz. Just over half of that time was spent processing the region from 1.45 ppm–1.02 ppm, highlighting that the time to perform estimation increases rapidly as the numbers of signals and data points present increase. If NMR-EsPy attracts sufficient interest, a future pursuit may be to improve the efficiency of the optimization routine, by employing a low-level language such as C++ or Rust, and exploiting concurrent programming.

## Conclusions

This article has demonstrated proof of principle for CUPID, a procedure for generating broadband pure shift spectra from 2DJ data sets by parametric estimation. The accompanying data and code allow the interested reader to reproduce the method; while the current implementation requires some user intervention, it should be relatively straightforward to produce fully automated code for routine use. CUPID can both generate absorption-mode pure shift spectra and extract the multiplet structure for each pure shift peak. Because it uses all of the available signal, CUPID can generate pure shift spectra where existing state-of-the-art broadband pure shift methods are too insensitive to provide usable spectra because of the signal loss incurred by the J-refocusing element. While space considerations preclude discussion in the current manuscript, in the longer term the CUPID approach promises to solve the long-standing problem of quantitation in pure shift NMR, and has the potential to greatly facilitate automated structural analysis.

## Supplementary Material



## Data Availability

10.5281/zenodo.17087135: Repository containing all the experimental and simulated NMR data sets presented in this work, including the Supporting Information, along with Jupyter notebooks used to generate the results.

## Notes

Document providing extra information about the following aspects of this work: 1.Theory underlying CUPID; 2.Additional information on data acquisition and result generation, and 3.Additional results, with both simulated and experimental data sets considered (PDF)

## References

[1] Aue W. P., Karhan J., Ernst R. (1976). Homonuclear broad band decoupling
and two-dimensional J-resolved NMR spectroscopy. J. Chem. Phys..

[2] Morris, G. A. eMagRes; Harris, R. K. ; Wasylishen, R. L. , Eds.; John Wiley & Sons, 2009 10.1002/9780470034590.

[3] Keeler J., Neuhaus D. (1985). Comparison and evaluation
of methods for two-dimensional
NMR spectra with absorption-mode lineshapes. J. Magn. Reson..

[4] Bax A., Freeman R., Morris G. A. (1981). A simple method for suppressing dispersion-mode
contributions in NMR spectra: The “pseudo echo”. J. Magn. Reson..

[5] Lindon J., Ferrige A. (1980). Digitisation and data processing
in Fourier transform
NMR. Prog. Nucl. Mag. Res. Sp..

[6] Zangger K. (2015). Pure shift
NMR. Prog. Nucl. Magn. Res. Spectrosc..

[7] Zangger K., Sterk H. (1997). Homonuclear Broadband-Decoupled
NMR Spectra. J. Magn. Reson..

[8] Aguilar J., Faulkner S., Nilsson M., Morris G. (2010). Pure Shift 1H NMR:
A Resolution of the Resolution Problem?. Angew.
Chem., Int. Ed..

[9] Garbow J., Weitekamp D., Pines A. (1982). Bilinear rotation decoupling of homonuclear
scalar interactions. Chem. Phys. Lett..

[10] Bax A. (1983). Broadband
homonuclear decoupling in heteronuclear shift correlation NMR spectroscopy. J. Magn. Reson..

[11] Foroozandeh M., Adams R. W., Meharry N. J., Jeannerat D., Nilsson M., Morris G. A. (2014). Ultrahigh-Resolution NMR Spectroscopy. Angew. Chem., Int. Ed..

[12] Foroozandeh M., Morris G. A., Nilsson M. (2018). PSYCHE Pure Shift NMR Spectroscopy. Chem. - Eur. J..

[13] Pell A. J., Keeler J. (2007). Two-dimensional J-spectra with absorption-mode lineshapes. J. Magn. Reson..

[14] Foroozandeh M., Adams R. W., Kiraly P., Nilsson M., Morris G. A. (2015). Measuring
couplings in crowded NMR spectra: pure shift NMR with multiplet analysis. Chem. Commun..

[15] Zhan H., Liu J., Fang Q., Chen X., Ni Y., Zhou L. (2024). Fast Pure
Shift NMR Spectroscopy Using Attention-Assisted Deep Neural Network. Adv. Sci..

[16] Zhan H., Liu J., Fang Q., Chen X., Hu L. (2024). Accelerated Pure Shift
NMR Spectroscopy with Deep Learning. Anal. Chem..

[17] Nuzillard J.-M. (1996). Time-Reversal
of NMR Signals by Linear Prediction. Application to Phase-Sensitive
HomonuclearJ-Resolved Spectroscopy. J. Magn.
Reson. A.

[18] Martinez A., Bourdreux F., Riguet E., Nuzillard J.-M. (2012). High-resolution
and high-sensitivity 2D homonuclear J-resolved NMR spectroscopy. Magn. Reson. Chem..

[19] Mutzenhardt P., Guenneau F., Canet D. (1999). A Procedure for Obtaining Pure Absorption
2D J-Spectra: Application to Quantitative Fully J-Decoupled Homonuclear
NMR Spectra. J. Magn. Reson..

[20] Mandelshtam V. A., Taylor H. S. (1997). Harmonic inversion
of time signals and its applications. J. Chem.
Phys..

[21] Mandelshtam V. A., Van Q. N., Shaka A. J. (1998). Obtaining Proton Chemical Shifts
and Multiplets from Several 1D NMR Signals. J. Am. Chem. Soc..

[22] Hulse, S. G. NMR Estimation in Python, 2025. https://github.com/foroozandehgroup/NMR-EsPy.

[23] Hulse S. G., Foroozandeh M. (2022). Newton meets
Ockham: Parameter estimation and model
selection of NMR data with NMR-EsPy. J. Magn.
Reson..

[24] Wax M., Kailath T. (1985). Detection
of signals by information theoretic criteria. IEEE Trans. Acoust., Speech, Signal Process..

[25] Lin Y.-Y., Hodgkinson P., Ernst M., Pines A. (1997). A Novel Detection-Estimation
Scheme for Noisy NMR Signals: Applications to Delayed Acquisition
Data. J. Magn. Reson..

[26] Hua Y. (1992). Estimating
two-dimensional frequencies by matrix enhancement and matrix pencil. IEEE Trans. Signal Process..

[27] Chen F. J., Fung C. C., Kok C. W., Kwong S. (2007). Estimation of two-dimensional
frequencies using modified matrix pencil method. IEEE Trans. Signal Process..

[28] Martini B. R., Mandelshtam V. A., Morris G. A., Colbourne A. A., Nilsson M. (2013). Filter diagonalization method for processing PFG NMR
data. J. Magn. Reson..

