# Supplementary material for: Full-Signal Ultrahigh-Resolution NMR by Parameter Estimation
Source: Anal Chem. 2025 Nov 8;97(45):25020–5. doi: 10.1021/acs.analchem.5c03446 (PMC12631727; doi:10.1021/acs.analchem.5c03446)
Supplement: Supplementary file 1 [file ac5c03446_si_001.pdf]

Supporting Information

Full-Signal Ultrahigh-Resolution NMR by Parameter Estimation

Simon G. Hulse[1], Mathias Nilsson[2], Gareth A. Morris[2], Mohammadali Foroozandeh[1,*]

[1] *Chemistry Research Laboratory, University of Oxford, 12 Mansfield Rd, Oxford, OX1 3TA, UK*
[2] *Department of Chemistry, University of Manchester, Oxford Road, Manchester M13 9PL, UK*

*mohammadali.foroozandeh@chem.ox.ac.uk

Further information on this work can be found in Chapters 2, 4 & 5 of the following thesis:

S. G. Hulse. "Estimation of NMR signals in the time domain: methodology, applications and software". PhD thesis. University of Oxford, 2023. DOI: 10.5287/ora-bpdg8rr9k

Contents

S1 Theory Overview **S7**

S1.1 The 2DJ Signal S7

S1.2 Newton's Method S7

S1.2.1 Model Derivatives S8

S1.2.2 Variance of Phases S10

S1.2.3 Approximating the Hessian S10

S1.2.4 Standard Errors S11

S1.3 Generating an Initial Guess S11

S1.4 Frequency Filtration S12

S1.5 The -45° Signal S14

S2 Methods **S14**

S2.1 Data acquisition S14

S2.1.1 2DJ experiments S14

S2.1.2 PSYCHE experiments S15

S2.1.3 Simulated datasets S15

S2.2 Result generation S16

S3 Extra Results **S17**

S3.1 Four multiplets S17

S3.2 Strychnine S17

S3.3 Camphor S17

S3.4 Dexamethasone S18

S4 Figures **S19**

S5 Tables **S30**

S6 Algorithms **S36**

S7 Code Listings S45

List of Figures

S1 PSYCHE pulse sequence used for the acquisition of estradiol data. S19

S2 TSE-PSYCHE pulse sequence used for the acquisition of dexamethasone data. . . . S20

S3 Overview of the frequency-filtration method applied to J-Resolved data. S21

S4 Illustration of the reasoning behind the name "−45° signal". S22

S5 Zoomed-in depiction of the quinine estimation result, focusing on the signals arising from proton (D) and water. S23

S6 Application of CUPID on simulated 2DJ datasets comprising four heavily overlapping ddd multiplet structures. S24

S7 Structures of strychnine, camphor and dexamethasone. S25

S8 Application of CUPID on a simulated strychnine 2DJ dataset. S26

S9 Application of CUPID on a 2DJ dataset of camphor. S27

S10 Application of CUPID on a 2DJ dataset of dexamethasone. S28

S11 Screenshots of the NMR-EsPy GUI. S29

List of Tables

S1 Experiment parameters for the 2DJ and PSYCHE experiments run. S30

S2 Chemical shifts and scalar couplings of spin systems used in Spinach simulations. . S31

S3 Metrics related to the results generated in this work. S33

List of Algorithms

S1 Trust-region numerical optimisation routine employed in NMR-EsPy. S36

S2 Steihaug-Toint method. S37

S3 MMEMPM. S38

S4 MDL. S41

S5 Frequency-filtration procedure. S42

S6 Proposed simplified frequency-filtration procedure. S43

S7 Multiplet grouping procedure. S44

List of Code Listings

S1 2DJ pulse sequence for use in Spinach. S45

S2 Python function used to transform a PSYCHE interferogram into a 1D FID. S46

Acronyms

1D one-dimensional

2D two-dimensional

2DJ two-dimensional J-Resolved

CPU central processing unit

CUPID computer-assisted undiminished-sensitivity protocol for ideal decoupling

DMSO-d_6 deuterated dimethyl sulfoxide

FID free induction decay

FT Fourier transform

GUI graphical user interface

IFT inverse Fourier transform

MDL minimum description length

MMEMPM modified matrix enhancement matrix pencil method

MPM matrix pencil method

NMR nuclear magnetic resonance

NMR-EsPy NMR Estimation in Python

PSYCHE pure shift yielded by chirp excitation

SVD singular value decomposition

TSE triple spin echo

Nomenclature

General Maths

$y := x$ — y is defined to be equal to x.

$\lfloor x \rfloor$ — The nearest integer to $x \in \mathbb{R}$ such that $\lfloor x \rfloor < x$.

$\lceil x \rceil$ — The nearest integer to $x \in \mathbb{R}$ such that $\lceil x \rceil > x$.

$\lfloor x \rceil$ — The nearest integer to $x \in \mathbb{R}$.

$\text{arctan2}(y, x)$ — The angle between the positive x-axis, and the line which passes through the origin and the point (x, y).

Sets

$x \in S$ — x is an element of the set S.

$S \cap T$ — Intersection of the sets S and T, i.e. the set of all elements which occur in both S and T.

$\mathbb{R}$ — Set of real numbers.

$\mathbb{R}_{>0}$ — Set of real numbers greater than 0.

$\mathbb{R}_{>1}$ — Set of real numbers greater than 1.

$\mathbb{C}$ — Set of complex numbers.

$\mathbb{Z}$ — Set of integers.

$\mathbb{N}_0$ — Set of natural numbers, with 0 included.

$[a, b]$ — Closed interval of a and b:

$$[a, b] := \{x \in \mathbb{R} | a \leq x \leq b\}.$$

$(a, b]$ — Half-open interval of a and b:

$$(a, b] := \{x \in \mathbb{R} | a < x \leq b\}.$$

$[[a, b]]$ — Discrete interval of a and b:

$$[[a, b]] := [a, b] \cap \mathbb{Z}$$

Vectors and Matrices

$\boldsymbol{a}$, $\boldsymbol{A}$, $a_{m,n}$ — Vectors are denoted by bold lower-case letters. Matrices are denoted by bold upper-case letters. Individual vector/matrix elements are denoted with lower-case letters, with subscript indices. For example the element of matrix $\boldsymbol{A}$ located at the 2nd row and 3rd column is denoted $a_{2,3}$.

$\langle \boldsymbol{a}, \boldsymbol{b} \rangle$, $\langle \boldsymbol{A}, \boldsymbol{B} \rangle$ — Euclidian inner product between two vectors $\boldsymbol{a}, \boldsymbol{b} \in \mathbb{C}^M$ or matrices $\boldsymbol{A}, \boldsymbol{B} \in \mathbb{C}^{M \times N}$:

$$\langle \boldsymbol{a}, \boldsymbol{b} \rangle := \sum_{m=1}^{M} a_m^* b_m \qquad \langle \boldsymbol{A}, \boldsymbol{B} \rangle := \sum_{m=1}^{M} \sum_{n=1}^{N} a_{m,n}^* b_{m,n}.$$

$\|\boldsymbol{a}\|$, $\|\boldsymbol{A}\|$ — The norm, defined as:

$$\|\boldsymbol{a}\| := \sqrt{\langle \boldsymbol{a}, \boldsymbol{a} \rangle} \qquad \|\boldsymbol{A}\| := \sqrt{\langle \boldsymbol{A}, \boldsymbol{A} \rangle}.$$

$\boldsymbol{A} \odot \boldsymbol{B}$ — The Hadamard (element-wise) product of $\boldsymbol{A}, \boldsymbol{B}$, two matrices with the same shape.

$\boldsymbol{a} \otimes \boldsymbol{b}$ — The outer product of $\boldsymbol{a} \in \mathbb{F}^m$ and $\boldsymbol{b} \in \mathbb{F}^n$, yielding the matrix $\boldsymbol{C} \in \mathbb{F}^{m \times n}$, with

$$c_{i,j} = a_i b_j$$

$\forall i \in [[1, m]]$, $\forall j \in [[1, n]]$.

Other Symbols

$\cdot^{(d)}$ — Dimension index. $d = 1$ refers to the indirect dimension of the 2DJ experiment/dataset, while $d = 2$ refers to the direct dimension.

$N^{(d)}$ — Number of points in dimension d.

$f_{\text{sw}}^{(d)}$ — Spectral width in dimension d (Hz).

f_{off} — Transmitter offset of the ^{1}H channel (Hz).

f_{sfo} — Transmitter frequency of the ^{1}H channel (MHz).

$\boldsymbol{\theta}^{(k)}$ — Parameter estimate at the k-th iteration of numerical optimisation.

$\boldsymbol{\theta}^{(0)}$ — Initial parameter estimate.

$\boldsymbol{\theta}^{(*)}$ — Parameter estimate at convergence.

S1 Theory Overview

S1.1 The 2DJ Signal

2DJ NMR experiments generate complex FIDs $\boldsymbol{Y} \in \mathbb{C}^{N^{(1)} \times N^{(2)}}$ which are assumed to obey the following form:

$$\boldsymbol{Y} = \boldsymbol{X}(\boldsymbol{\theta}) + \boldsymbol{W}, \tag{1a}$$

$$\begin{aligned} x_{n^{(1)},n^{(2)}}(\boldsymbol{\theta}) = \sum_{m=1}^{M} a_m \exp(\mathrm{i}\phi_m) \exp\left[\left(2\pi\mathrm{i} f_m^{(1)} - \eta_m^{(1)}\right)\left(n^{(1)} - 1\right)\tau^{(1)}\right] \\ \times \exp\left[\left(2\pi\mathrm{i}\left(f_m^{(2)} - f_{\text{off}}\right) - \eta_m^{(2)}\right)\left(n^{(2)} - 1\right)\tau^{(2)}\right], \end{aligned} \tag{1b}$$

$$\mathbb{R}^{6M} \ni \boldsymbol{\theta} = \begin{bmatrix} a_1 & \cdots & a_M & \phi_1 & \cdots & \phi_M & f_1^{(1)} & \cdots & f_M^{(1)} & \cdots & f_1^{(2)} & \cdots & \eta_1^{(1)} & \cdots & \eta_1^{(2)} & \cdots & \eta_M^{(2)} \end{bmatrix}^{\mathrm{T}}, \tag{1c}$$

where $\boldsymbol{W}$ is a matrix of experimental noise, $n^{(1/2)} \in [[1, N^{(1/2)}]]$, and $\tau^{(1/2)} := 1/f_{\text{sw}}^{(1/2)}$ is the dwell time in dimension (1/2).

The parameter vector $\boldsymbol{\theta}$ comprises the following elements, which describe the FID's M constituent signals:

- Amplitude $a_m \in \mathbb{R}_{>0}$,
- Phase $\phi_m \in (-\pi, \pi]$,
- Frequency in the indirect dimension $f_m^{(1)} \in [-f_{\text{sw}}^{(1)}/2,\ f_{\text{sw}}^{(1)}/2]$,
- Frequency in the direct dimension $f_m^{(2)} \in [f_{\text{off}} - f_{\text{sw}}^{(2)}/2, f_{\text{off}} + f_{\text{sw}}^{(2)}/2]$
- Damping factor in each dimension $\eta_m^{(1/2)} \in \mathbb{R}_{>0}$

S1.2 Newton's Method

The method used in NMR-EsPy to estimate $\boldsymbol{\theta}$ closely resembles the 1D method that has been presented previously [1, Chapter 2; 2]. In brief, a parsimonious set of parameters is sought which minimises the cost function $\mathcal{F}(\boldsymbol{\theta}|\boldsymbol{Y}) : \mathbb{C}^{N^{(1)} \times N^{(2)}} \times \mathbb{R}^{6M} \to \mathbb{R}$, defined as

$$\mathcal{F}(\boldsymbol{\theta}|\boldsymbol{Y}) = \|\boldsymbol{Y} - \boldsymbol{X}(\boldsymbol{\theta})\|^2 + \mathrm{Var}(\boldsymbol{\phi}) \equiv \langle \boldsymbol{Y} - \boldsymbol{X}(\boldsymbol{\theta}), \boldsymbol{Y} - \boldsymbol{X}(\boldsymbol{\theta}) \rangle + \mathrm{Var}(\boldsymbol{\phi}). \tag{2}$$

$\mathrm{Var}(\boldsymbol{\phi})$ denotes the *circular* variance of signal phases (*vide infra*). The cost function is minimised using numerical optimisation; specifically, the method adopted here is a trust-region algorithm, using a truncated conjugate-gradient approach commonly called the Steihaug-Toint method [3,

Chapters 4 & 7] (see also Algorithms S1 and S2, Pages S36 and S37). The routine approximates the cost function as quadratic about the parameter estimate at each iteration k, and therefore the gradient $\nabla\mathcal{F}(\boldsymbol{\theta}^{(k)}|\boldsymbol{Y}) \in \mathbb{R}^{6M}$ and Hessian $\nabla^2\mathcal{F}(\boldsymbol{\theta}^{(k)}|\boldsymbol{Y}) \in \mathbb{R}^{6M\times 6M}$ of the cost function are required:

$$\mathcal{F}_{\mathrm{Q}}(\boldsymbol{\theta}|\boldsymbol{Y}) = \mathcal{F}(\boldsymbol{\theta}^{(k)}|\boldsymbol{Y}) + \boldsymbol{h}^{\mathrm{T}}\nabla\mathcal{F}(\boldsymbol{\theta}^{(k)}|\boldsymbol{Y}) + \frac{1}{2}\boldsymbol{h}^{\mathrm{T}}\nabla^2\mathcal{F}(\boldsymbol{\theta}^{(k)}|\boldsymbol{Y})\boldsymbol{h}, \tag{3a}$$

$$\left[\nabla\mathcal{F}(\boldsymbol{\theta}|\boldsymbol{Y})\right]_i = -2\,\mathrm{Re}\left\langle \boldsymbol{Y} - \boldsymbol{X}(\boldsymbol{\theta}), \frac{\partial \boldsymbol{X}(\boldsymbol{\theta})}{\partial\theta_i}\right\rangle + \frac{\partial\,\mathrm{Var}(\boldsymbol{\phi})}{\partial\theta_i}, \tag{3b}$$

$$\left[\nabla^2\mathcal{F}(\boldsymbol{\theta}|\boldsymbol{Y})\right]_{i,j} = 2\,\mathrm{Re}\left(\left\langle \frac{\partial \boldsymbol{X}(\boldsymbol{\theta})}{\partial\theta_i}, \frac{\partial \boldsymbol{X}(\boldsymbol{\theta})}{\partial\theta_j}\right\rangle - \left\langle \boldsymbol{Y} - \boldsymbol{X}(\boldsymbol{\theta}), \frac{\partial^2 \boldsymbol{X}(\boldsymbol{\theta})}{\partial\theta_i\partial\theta_j}\right\rangle\right) + \frac{\partial^2\,\mathrm{Var}(\boldsymbol{\phi})}{\partial\theta_i\partial\theta_j}, \tag{3c}$$

$\forall i,j \in [[1, 6M]]$, with $\boldsymbol{h} = \boldsymbol{\theta} - \boldsymbol{\theta}^{(k)}$ and

$$\frac{\partial \boldsymbol{X}(\boldsymbol{\theta})}{\partial\theta_i} := \begin{bmatrix} \frac{\partial x_{1,1}}{\partial\theta_i} & \frac{\partial x_{1,2}}{\partial\theta_i} & \cdots & \frac{\partial x_{1,N^{(2)}}}{\partial\theta_i} \\ \frac{\partial x_{2,1}}{\partial\theta_i} & \frac{\partial x_{2,2}}{\partial\theta_i} & \cdots & \frac{\partial x_{2,N^{(2)}}}{\partial\theta_i} \\ \vdots & \vdots & \ddots & \vdots \\ \frac{\partial x_{N^{(1)},1}}{\partial\theta_i} & \frac{\partial x_{N^{(1)},2}}{\partial\theta_i} & \cdots & \frac{\partial x_{N^{(1)},N^{(2)}}}{\partial\theta_i} \end{bmatrix}, \tag{4a}$$

$$\frac{\partial^2 \boldsymbol{X}(\boldsymbol{\theta})}{\partial\theta_i\partial\theta_j} := \begin{bmatrix} \frac{\partial^2 x_{1,1}}{\partial\theta_i\partial\theta_j} & \frac{\partial^2 x_{1,2}}{\partial\theta_i\partial\theta_j} & \cdots & \frac{\partial^2 x_{1,N^{(2)}}}{\partial\theta_i\partial\theta_j} \\ \frac{\partial^2 x_{2,1}}{\partial\theta_i\partial\theta_j} & \frac{\partial^2 x_{2,2}}{\partial\theta_i\partial\theta_j} & \cdots & \frac{\partial^2 x_{2,N^{(2)}}}{\partial\theta_i\partial\theta_j} \\ \vdots & \vdots & \ddots & \vdots \\ \frac{\partial^2 x_{N^{(1)},1}}{\partial\theta_i\partial\theta_j} & \frac{\partial^2 x_{N^{(1)},2}}{\partial\theta_i\partial\theta_j} & \cdots & \frac{\partial^2 x_{N^{(1)},N^{(2)}}}{\partial\theta_i\partial\theta_j} \end{bmatrix}. \tag{4b}$$

S1.2.1 Model Derivatives

The complete set of expressions for all first and second derivatives of the model elements $x_{n^{(1)},n^{(2)}}$ are as follows $\forall n^{(1/2)} \in [[1, N^{(1/2)}]]$, $\forall m \in [[1, M]]$, $\forall i,j \in [[1, 6M]]$, $\forall d, d' \in \{1, 2\}$:

First derivatives

$$\frac{\partial x_{n^{(1)},n^{(2)}}}{\partial\theta_m} \equiv \frac{\partial x_{n^{(1)},n^{(2)}}}{\partial a_m} = \frac{x_{n^{(1)},n^{(2)}}}{a_m}, \tag{5a}$$

$$\frac{\partial x_{n^{(1)},n^{(2)}}}{\partial\theta_{m+M}} \equiv \frac{\partial x_{n^{(1)},n^{(2)}}}{\partial\phi_m} = \mathrm{i}x_{n^{(1)},n^{(2)}}, \tag{5b}$$

$$\frac{\partial x_{n^{(1)},n^{(2)}}}{\partial \theta_{m+(d+1)M}} \equiv \frac{\partial x_{n^{(1)},n^{(2)}}}{\partial f_m^{(d)}} = 2\pi \mathrm{i} \tau^{(d)} n^{(d)} x_{n^{(1)},n^{(2)}}, \tag{5c}$$

$$\frac{\partial x_{n^{(1)},n^{(2)}}}{\partial \theta_{m+(d+3)M}} \equiv \frac{\partial x_{n^{(1)},n^{(2)}}}{\partial \eta_m^{(d)}} = -\tau^{(d)} n^{(d)} x_{n^{(1)},n^{(2)}}. \tag{5d}$$

Second Derivatives

$$\frac{\partial^2 x_{n^{(1)},n^{(2)}}}{\partial \theta_m^2} \equiv \frac{\partial^2 x_{n^{(1)},n^{(2)}}}{\partial a_m^2} = 0, \tag{6a}$$

$$\frac{\partial^2 x_{n^{(1)},n^{(2)}}}{\partial \theta_m \partial \theta_{m+M}} \equiv \frac{\partial^2 x_{n^{(1)},n^{(2)}}}{\partial a_m \partial \phi_m} = \frac{\mathrm{i} x_{n^{(1)},n^{(2)}}}{a_m}, \tag{6b}$$

$$\frac{\partial^2 x_{n^{(1)},n^{(2)}}}{\partial \theta_m \partial \theta_{m+(d+1)M}} \equiv \frac{\partial^2 x_{n^{(1)},n^{(2)}}}{\partial a_m \partial f_m^{(d)}} = \frac{2\pi \mathrm{i} \tau^{(d)} n^{(d)} x_{n^{(1)},n^{(2)}}}{a_m}, \tag{6c}$$

$$\frac{\partial^2 x_{n^{(1)},n^{(2)}}}{\partial \theta_m \partial \theta_{m+(d+3)M}} \equiv \frac{\partial^2 x_{n^{(1)},n^{(2)}}}{\partial a_m \partial \eta_m^{(d)}} = \frac{-\tau^{(d)} n^{(d)} x_{n^{(1)},n^{(2)}}}{a_m}, \tag{6d}$$

$$\frac{\partial^2 x_{n^{(1)},n^{(2)}}}{\partial \theta_{m+M}^2} \equiv \frac{\partial^2 x_{n^{(1)},n^{(2)}}}{\partial \phi_m^2} = -x_{n^{(1)},n^{(2)}}, \tag{6e}$$

$$\frac{\partial^2 x_{n^{(1)},n^{(2)}}}{\partial \theta_{m+M} \partial \theta_{m+(d+1)M}} \equiv \frac{\partial^2 x_{n^{(1)},n^{(2)}}}{\partial \phi_m \partial f_m^{(d)}} = -2\pi \tau^{(d)} n^{(d)} x_{n^{(1)},n^{(2)}}, \tag{6f}$$

$$\frac{\partial^2 x_{n^{(1)},n^{(2)}}}{\partial \theta_{m+M} \partial \theta_{m+(d+3)M}} \equiv \frac{\partial^2 x_{n^{(1)},n^{(2)}}}{\partial \phi_m \partial \eta_m^{(d)}} = -\mathrm{i} \tau^{(d)} n^{(d)} x_{n^{(1)},n^{(2)}}, \tag{6g}$$

$$\frac{\partial^2 x_{n^{(1)},n^{(2)}}}{\partial \theta_{m+(d+1)M} \partial \theta_{m+(d'+1)M}} \equiv \frac{\partial^2 x_{n^{(1)},n^{(2)}}}{\partial f_m^{(d)} \partial f_m^{(d')}} = -4\pi^2 \left(\tau^{(d)} n^{(d)}\right) \left(\tau^{(d')} n^{(d')}\right) x_{n^{(1)},n^{(2)}}, \tag{6h}$$

$$\frac{\partial^2 x_{n^{(1)},n^{(2)}}}{\partial \theta_{m+(d+1)M} \partial \theta_{m+(d'+3)M}} \equiv \frac{\partial^2 x_{n^{(1)},n^{(2)}}}{\partial f_m^{(d)} \partial \eta_m^{(d')}} = -2\pi \mathrm{i} \left(\tau^{(d)} n^{(d)}\right) \left(\tau^{(d')} n^{(d')}\right) x_{n^{(1)},n^{(2)}}, \tag{6i}$$

$$\frac{\partial^2 x_{n^{(1)},n^{(2)}}}{\partial \theta_{m+(d+3)M} \partial \theta_{m+(d'+3)M}} \equiv \frac{\partial^2 x_{n^{(1)},n^{(2)}}}{\partial \eta_m^{(d)} \partial \eta_m^{(d')}} = \left(\tau^{(d)} n^{(d)}\right) \left(\tau^{(d')} n^{(d')}\right) x_{n^{(1)},n^{(2)}}, \tag{6j}$$

$$\frac{\partial^2 x_{n^{(1)},n^{(2)}}}{\partial \theta_i \partial \theta_j} = \frac{\partial^2 x_{n^{(1)},n^{(2)}}}{\partial \theta_j \partial \theta_i}, \tag{6k}$$

$$\frac{\partial^2 x_{n^{(1)},n^{(2)}}}{\partial \theta_i \partial \theta_j} = 0 \quad \text{if not specified above.} \tag{6l}$$

S1.2.2 Variance of Phases

Oscillator phases are an example of a *circular variable*, since all values are periodic within the interval $(-\pi, \pi]$. It is necessary that a definition of variance which accounts for this periodicity is used. The circular variance is defined as [4, Chapter 3]

$$[0,1] \ni \operatorname{Var}(\boldsymbol{\phi}) = 1 - \frac{R}{M}, \tag{7a}$$

$$R = \sqrt{c_\Sigma^2 + s_\Sigma^2}, \tag{7b}$$

$$c_\Sigma = \sum_{m=1}^{M} \cos\phi_m, \tag{7c}$$

$$s_\Sigma = \sum_{m=1}^{M} \sin\phi_m. \tag{7d}$$

The first and second derivatives of the circular variance are given by

$$\frac{\partial \operatorname{Var}(\boldsymbol{\phi})}{\partial\theta_i} = \begin{cases} \dfrac{\xi_i}{MR} & M < i \leq 2M \\ 0 & \text{otherwise} \end{cases} \tag{8a}$$

$$\frac{\partial^2 \operatorname{Var}(\boldsymbol{\phi})}{\partial\theta_i \partial\theta_j} = \begin{cases} \dfrac{1}{RM}\left(\dfrac{\xi_i^2}{R^2} + \cos\phi_{i-M} c_\Sigma + \sin\phi_{i-M} s_\Sigma - 1\right) & M < i,j \leq 2M, i = j \\ \dfrac{1}{RM}\left(\dfrac{\xi_i\xi_j}{R^2} - \cos\left(\phi_{i-M} - \phi_{j-M}\right)\right) & M < i,j \leq 2M, i \neq j \\ 0 & \text{otherwise} \end{cases}, \tag{8b}$$

$$\xi_i = \sin\phi_{i-M} c_\Sigma - \cos\phi_{i-M} s_\Sigma. \tag{8c}$$

S1.2.3 Approximating the Hessian

Computation of the Hessian matrix (Equation 3c) is expensive, with one of the most demanding aspects being generating all the $21MN^{(1)}N^{(2)}$ second derivatives required [1, Section 2.4.2]. The default behaviour in NMR-EsPy is to approximate the Hessian matrix by neglecting the term involving model second derivatives, i.e.

$$\left[\nabla^2 \mathcal{F}(\boldsymbol{\theta}|\boldsymbol{Y})\right]_{i,j} = 2\operatorname{Re}\left\langle \frac{\partial \boldsymbol{X}(\boldsymbol{\theta})}{\partial\theta_i}, \frac{\partial \boldsymbol{X}(\boldsymbol{\theta})}{\partial\theta_j} \right\rangle + \frac{\partial^2 \operatorname{Var}(\boldsymbol{\phi})}{\partial\theta_i\partial\theta_j}, \tag{9}$$

While more iterations are typically necessary for the optimiser to reach convergence when this approximation is invoked, each iteration is considerably faster than its exact counterpart. The total time required to converge is often far shorter for this reason.

S1.2.4 Standard Errors

The standard errors associated with each parameter in the final result $\boldsymbol{\theta}^{(*)}$ can be estimated using [1, Section A.2.2; 5, Section 2.7]

$$\boldsymbol{\epsilon}\left(\boldsymbol{\theta}^{(*)}\right) = \sqrt{\frac{\mathcal{F}(\boldsymbol{\theta}^{(*)}|\boldsymbol{Y})\,\mathrm{diag}\left(\left[\nabla^2\mathcal{F}(\boldsymbol{\theta}^{(*)}|\boldsymbol{Y})\right]^{-1}\right)}{N^{(1)}N^{(2)}-1}}. \tag{10}$$

S1.3 Generating an Initial Guess

Numerical optimisation requires the specification of an initial parameter guess $\boldsymbol{\theta}^{(0)}$ which is sufficiently close to a (local) minimum of the cost function for successful application. In NMR-EsPy, the MMEMPM — an extension of the 1D MPM [6] for 2D data — is employed [1, Section 2.2.2; 7; 8] (see Algorithm S3). One of the central steps in the MMEMPM is the application of SVD on a block-Hankel matrix featuring the points in $\boldsymbol{Y}$ (Lines 2 to 7 in Algorithm S3). Applying a complete SVD on such a large matrix — with approximate size $N^{(1)}N^{(2)}/4 \times N^{(1)}N^{(2)}/4$ — is extremely computationally expensive. Given that it is only necessary to determine the M most significant components of the SVD (for typical 2DJ datasets, $M \ll N^{(1)}N^{(2)}/4$), it is more efficient to compute the *truncated* SVD of the block-Hankel matrix, by using an iterative approach such as the Arnoldi iteration [9]. This is provided by the `scipy.sparse.linalg.svds` function present in the SciPy library [10].

Prior to applying the MMEMPM, an estimate of the number of constituent signals M in the 2DJ FID is required. It is desirable to predict M using an unbiased criterion that doesn't rely on user intervention. The MDL criterion has found wide favour for use in signal processing applications [11; 12; 13]. It would be ideal to apply the MDL on the entire 2DJ dataset. However, this would require a complete SVD to be performed on the large block-Hankel matrix (*vide supra*). As an inferior though more efficient alternative, an estimate of M can be obtained by applying the MDL on the first direct-dimension FID in the dataset, as outlined in Algorithm S4. This 1D FID is equivalent to an FID produced using a simple pulse-acquire experiment (consider Equation 1b with $n^{(1)} = 1$). Signals with very similar frequencies in the direct dimension — regardless of the extent of their separation in the indirect dimension — will be heavily overlapping and hard to resolve when the MDL is applied on the 1D FID. In cases where the 2DJ dataset is so densely populated with signals that applying this approach is ineffectual (the 17β-estradiol example an example) the user is able to provide a hard-coded prediction of M as a fallback.

One serendipitous feature in using the first direct-dimension FID for model order prediction relates to the impact on strong coupling artefacts. In pulse-acquire datasets, the effect of strong coupling is to augment particular signals; this phenomenon is commonly referred to as "roofing". Crucially, distinct signals do not manifest in pulse-acquire datasets on account of strong coupling effects, as they do in 2DJ datasets. As a result, the MDL often returns predictions of M which agree with

the number of first-order* signals in the 2DJ dataset, rather than the combined total of first-order signals and strong coupling artefacts. Since strong coupling artefacts tend to be lower in intensity, application of the MMEMPM — which provides a prediction of the M most significant components in the data — often leads to these being neglected from the final parameter estimate, such that their influence is suppressed in the final pure shift spectrum. It should be appreciated that this concept is not infallible; there are results presented here where strong coupling artefacts were incorporated into the final parameter estimate.

S1.4 Frequency Filtration

N.B. *The filtering procedure described in this section was used in the generation of all results presented in this work. However in retrospect, a more straightforward method of applying a rectangular filter, and slicing the filtered spectrum exactly at the filter boundaries would likely yield sub-FIDs with the same information content, without the requirement to incorporate synthetic noise into the filtered dataset. See Algorithm S6 for details.*

Application of the described method on complete 2DJ signals is very computationally expensive. The features which have the greatest influence on computational cost are the number of datapoints the FID comprises ($N^{(1)}$ and $N^{(2)}$), and the number of oscillators being used to parameterise the FID (M); a detailed illustration of this is provided in [1, Section 2.4]. These can both be reduced considerably by breaking the full FID into a series frequency-filtered sub-FIDs, comprising the regions that are of interest to the user. For 2DJ datasets, filtering is only applied in the direct dimension, as the indirect dimension tends to be densely populated. The filtration procedure used in NMR-EsPy involves the following steps:

1. Formation of a *virtual echo* $\hat{\boldsymbol{Y}} \in \mathbb{C}^{N^{(1)} \times 2N^{(2)}}$ along the direct dimension[15; 16]:

$$\hat{\boldsymbol{y}}_{n^{(1)}} = \left[\operatorname{Re}\left(y_{n^{(1)},1}\right)\ y_{n^{(1)},2}\ \cdots\ y_{n^{(1)},N^{(2)}}\ 0\ y^*_{n^{(1)},N^{(2)}}\ y^*_{n^{(1)},N^{(2)}-1}\ \cdots\ y^*_{n^{(1)},2}\right] \tag{11}$$

2. FT of $\hat{\boldsymbol{Y}}$ in the direct dimension, to yield $\hat{\boldsymbol{S}} \in \mathbb{R}^{N^{(1)} \times 2N^{(2)}}$. By design, the FT of a virtual echo yields a spectrum with an imaginary component of zeros, which is discarded.

3. Application of a super-Gaussian filter, and the addition of Gaussian White noise, whose variance is dependent on the magntiude of the filter at each point (see Figure S3):

$$\tilde{\boldsymbol{S}} = \hat{\boldsymbol{S}} \odot \boldsymbol{G} + \boldsymbol{W}_{\sigma^2} \odot (\boldsymbol{1} - \boldsymbol{G}), \tag{12a}$$

$$g_{n^{(1)},n^{(2)}} = \exp\left(-2^{p+1}\left(\frac{n^{(2)} - c}{b}\right)^p\right), \tag{12b}$$

*The term *first-order* is used here to refer to signals that are expected to be present in a 2DJ dataset when the weak-coupling approximation is invoked [14, Sections 14.5.1 & 17.6].

$\forall n^{(1)} \in [[1, N^{(1)}]]$, $\forall n^{(2)} \in [[1, 2N^{(2)}]]$. $\boldsymbol{W}_{\sigma^2} \in \mathbb{R}^{N^{(1)} \times 2N^{(2)}}$ is a matrix of independent random samples from a normal distribution of mean 0 and variance σ^2, with σ^2 being the noise variance of $\hat{\boldsymbol{S}}$. The noise variance is obtained by extracting the variance of a region in $\hat{\boldsymbol{S}}$ in which no discernible signals exist. $c \in [[1, 2N^{(2)}]]$ is the index of the center of the region to be filtered. $b \in [[1, 2N^{(2)}]]$ is the bandwidth of the filter (i.e. the number of points the filter region spans). $p \in \mathbb{R}_{>0}$ is a factor which dictates the steepness of the filter's edges; larger values of p make the super-Gaussian more box-like. This has been set to 40 for all results in this paper, and it is the default in NMR-EsPy.

c and b need to be derived from the left and right bounds of the spectral region of interest which will typically be given in ppm: $(l_{\text{ppm}}, r_{\text{ppm}})$. Conversion from ppm ($f_{\text{ppm}}$) to the corresponding spectrum index (f_{idx}) is achieved through

$$f_{\text{idx}} = \left\lfloor \frac{\left(N^{(2)} - 1\right)\left(f_{\text{sw}}^{(2)} + 2(f_{\text{off}} - f_{\text{sfo}} f_{\text{ppm}})\right)}{f_{\text{sw}}^{(2)}} \right\rceil \quad \forall f_{\text{ppm}} \in \left[\frac{f_{\text{off}} - (f_{\text{sw}}^{(2)}/2)}{f_{\text{sfo}}}, \frac{f_{\text{off}} + (f_{\text{sw}}^{(2)}/2)}{f_{\text{sfo}}}\right] \tag{13}$$

Assuming $r_{\text{ppm}} < l_{\text{ppm}}$, c and b are given by

$$c = \frac{l_{\text{idx}} + r_{\text{idx}}}{2} \tag{14a}$$

$$b = r_{\text{idx}} - l_{\text{idx}}. \tag{14b}$$

4. Removal of regions either side of the region of interest. This reduces the number of data points the sub-FID comprises while retaining the same information about the region of interest. Using the parameter $\gamma > 1$, spectral indices at which slicing is set to take place are given by

$$\lambda = \begin{cases} c - \left\lfloor \frac{\gamma b}{2} \right\rfloor & \text{if } \geq 1 \\ 1 & \text{otherwise} \end{cases}, \tag{15a}$$

$$\rho = \begin{cases} c + \left\lceil \frac{\gamma b}{2} \right\rceil & \text{if } \leq 2N^{(2)} \\ 2N^{(2)} & \text{otherwise} \end{cases}. \tag{15b}$$

A truncated spectrum $\bar{\boldsymbol{S}} \in \mathbb{R}^{N^{(1)} \times (\rho - \lambda)}$ is then created from $\tilde{\boldsymbol{S}}$ according to

$$\bar{s}_{n^{(1)}, n^{(2)}} = \tilde{s}_{n^{(1)}, n^{(2)} + \lambda}, \tag{16}$$

$\forall n^{(1)} \in [[1, N^{(1)}]]$, $\forall n^{(2)} \in [[1, \rho - \lambda]]$.

5. IFT of $\bar{\boldsymbol{S}}$, followed by scaling to account for the slicing in Item 4. The IFT on a real spectrum produces a conjugate symmetric time-domain signal; this is truncated to the first half of points

to yield the final sub-FID $\tilde{\boldsymbol{Y}} \in \mathbb{C}^{N^{(1)} \times \lfloor (\rho - \lambda)/2 \rfloor}$:

$$\tilde{y}_{n^{(1)},n^{(2)}} = \frac{\rho - \lambda}{N^{(2)}} \left[\mathrm{IFT} \left(\bar{\boldsymbol{S}} \right) \right]_{n^{(1)},n^{(2)}}, \tag{17}$$

$\forall n^{(1)} \in [[1, N^{(1)}]]$, $\forall n^{(2)} \in [[1, \lfloor (\rho - \lambda)/2 \rfloor]]$.

This filtering procedure is outlined in Algorithm S5. It is important to note that for accurate determination of the sub-FID's parameters, the effective spectral width and transmitter offset must be determined (these feature in Equation 1b):

$$\bar{f}_{\text{off}} = f_{\text{off}} + \frac{f_{\text{sw}}^{(2)}}{2} \left(1 - \frac{\rho - \lambda}{2N^{(2)}} \right), \tag{18a}$$

$$\bar{f}_{\text{sw}}^{(2)} = \frac{\rho - \lambda - 1}{N^{(2)} - 1} f_{\text{sw}}^{(2)}. \tag{18b}$$

S1.5 The -45° Signal

As described in the main text, CUPID yields a pure shift spectrum from the result of 2DJ estimation by constructing the $-45°$ signal $\boldsymbol{y}^{-45°} \in \mathbb{C}^{N^{(2)}}$:

$$y_n^{-45°}(\boldsymbol{\theta}) = \sum_{m=1}^{M} a_m \exp\left(\mathrm{i}\phi_m\right) \exp\left(\left(2\pi \mathrm{i} \left(f_m^{(2)} - f_m^{(1)} - f_{\text{off}} \right) - \eta_m^{(2)} \right) (n-1) \tau^{(2)} \right), \tag{19}$$

$\forall n \in [[1, N^{(2)}]]$. FT of $\boldsymbol{y}^{-45°}$ then leads to the pure shift spectrum. The reasoning behind the name is illustrated by Figure S4, Page S22. A pure shift spectrum can be generated by back-propagating a hypothetical 2DJ FID with $f_{\text{sw}}^{(1)} = f_{\text{sw}}^{(2)}$ and $N^{(1)} = N^{(2)}$ into negative $\tau^{(1)}$, and extracting the main diagonal. There is a slight discrepancy between this signal and $\boldsymbol{y}^{-45°}$, in that the contribution from damping in the indirect dimension, $\{\eta_m^{(1)}\}$, is neglected in Equation 19.

S2 Methods

S2.1 Data acquisition

S2.1.1 2DJ experiments

Four experimental 2DJ datasets are presented in this work, with two in the main text (quinine, estradiol), and two in the SI, of camphor and 39 mM dexamethasone, both dissolved in DMSO-d_6. The datasets were acquired on various BRUKER spectrometers. In each case, `jresqf`, part of BRUKER's standard pulse sequence library, was used. This comprises: $90°(\Phi_1) \rightarrow {}^{t^{(1)}}\!/2 \rightarrow 180°(\Phi_2) \rightarrow {}^{t^{(1)}}\!/2 \rightarrow$

$t^{(2)}(\Phi_{\mathrm{rx}})$, with the EXORCYCLE phase-cycling scheme [17]:

$$\begin{array}{lllll} \Phi_1: & 0° & 0° & 0° & 0° \\ \Phi_2: & 0° & 90° & 180° & 270° \\ \Phi_{\mathrm{rx}}: & 0° & 180° & 0° & 180° \end{array}$$

Noteworthy experimental parameters are provided in Table S1, Page S30.

Spectra were formed by applying sine-bell apodisation, FT in both dimensions, and finally computing the absolute value (magnitude) of each complex point. 45°-tilt spectra of quinine (Figure 2.a of the main text) and camphor (Figure S9.a) were produced by applying a right circular shift to the points in the 2DJ spectra; the tilted spectrum $\boldsymbol{T} \in \mathbb{R}^{N^{(1)} \times N^{(2)}}$ is related to the 2DJ spectrum $\boldsymbol{S} \in \mathbb{R}^{N^{(1)} \times N^{(2)}}$ as follows:

$$t_{n^{(1)},n^{(2)}} = s_{n^{(1)},n^{(2)\prime}} \tag{20a}$$

$$n^{(2)\prime} = \left(n^{(2)} + \left\lfloor \frac{f_{\mathrm{sw}}^{(1)} N^{(2)}}{f_{\mathrm{sw}}^{(2)} N^{(1)}} \left(\frac{N^{(1)}}{2} - n^{(1)}\right) \right\rfloor \right) \bmod N^{(2)}. \tag{20b}$$

S2.1.2 PSYCHE experiments

The pulse sequence used for acquisition of the estradiol PSYCHE spectrum (Figure 3.a of the main paper) is presented in Figure S1, Page S19, where pulses, gradients, and delays are described in detail. A pure shift spectrum was also acquired to accompany the dexamethasone example, using the TSE-PSYCHE experiment [18]. A description of the pulse sequence is provided in Figure S2, Page S20.

The 2D PSYCHE interferograms were transformed to 1D pure shift FIDs using the Python script shown in Code Listing S2, Page S46.

S2.1.3 Simulated datasets

Results formed by considering simulated 2DJ datasets are also presented here. All 2DJ simulations were performed using the Spinach Matlab library [19]. Many standard NMR experiments can be simulated out-of-the-box in Spinach using one of the functions provided in the `experiments/` directory. There isn't one provided for the 2DJ experiment however, so an in-house function was written for it, which is shown in Code Listing S1, Page S45.

Four Multiplets

A series of 5 2DJ datasets were simulated such that part of the spectrum comprised four heavily overlapping ddd multiplet structures. Spin systems with 7 spins were constructed, with the spins

separated into two different categories: 4 *estimated spins* (A) – (D) and 3 *coupling spins* (E) – (G). The estimated spins were assigned resonance frequencies sampled randomly from a uniform distribution with bounds −15 Hz to 15 Hz. Each of the estimated spins was coupled to each of the coupling spins, with the J-values randomly sampled from a uniform distribution with bounds 1 Hz to 10 Hz. Restrictions were placed on the shifts and couplings to ensure that

1. no two estimated spin resonance frequencies were separated by less than $f_{\text{sw}}^{(2)}/N^{(2)}$ Hz,
2. each multiplet structure did not comprise any resonances separated by less than $f_{\text{sw}}^{(1)}/N^{(1)}$ Hz.

The coupling spins were given resonance frequencies such that they lay far from the estimated spins in the spectrum, to prevent the presence of strong coupling artefacts. The resonance frequencies and couplings associated with the estimated spins are listed in Table S2, Page S31. Spin relaxation was simulated by employing Redfield theory, assuming spherical isotropic motion with autocorrelation time $\tau_{\text{c}} = 200\,\text{ps}$. Relevant experimental parameters provided to the simulations include: $f_{\text{sfo}} = 500\,\text{MHz}$; $f_{\text{sw}}^{(1)} = 40\,\text{Hz}$; $f_{\text{sw}}^{(2)} = 1000\,\text{Hz}$ (2 ppm); $f_{\text{off}} = 0\,\text{Hz}$; $N^{(1)} = 128$; $N^{(2)} = 1024$. Prior to estimation, each FID was filtered using the process described in Section S1.4, using bounds of 35 Hz to −35 Hz.

Strychnine

The chemical shifts and scalar couplings associated with strychnine, defined in Spinach in the file `etc/strychnine.m` were used to generate another simulated 2DJ dataset. See Table S2 for a outline of the chemical shifts and scalar couplings which contribute. Again, relaxation was simulated by applying Redfield theory and assuming spherical isotropic motion, with $\tau_{\text{c}} = 200\,\text{ps}$. Relevant experimental parameters provided to the simulation include: $f_{\text{sfo}} = 500\,\text{MHz}$; $f_{\text{sw}}^{(1)} = 50\,\text{Hz}$; $f_{\text{sw}}^{(2)} = 5000\,\text{Hz}$ (10 ppm); $f_{\text{off}} = 2500\,\text{Hz}$; $N^{(1)} = 128$; $N^{(2)} = 8192$.

S2.2 Result generation

All the results generated in this work used the NMR-EsPy Python package (version 2.1.0). Useful links related to the package include:

Source code https://github.com/foroozandehgroup/NMR-EsPy

Documentation homepage https://foroozandehgroup.github.io/NMR-EsPy/

Installation instructions https://foroozandehgroup.github.io/NMR-EsPy/install.html

Tutorial https://foroozandehgroup.github.io/NMR-EsPy/walkthroughs/estimator2dj.html

API https://foroozandehgroup.github.io/NMR-EsPy/references/estimator2dj.html

All results were produced using Jupyter Notebooks, which can be found within the `code/` directory at the DOI 10.5281/zenodo.17087135. The results were generated on a workstation with an Intel® Core™ i9-7920CPU @ 2.9 GHz. Useful metrics for each result, including timings, are presented in Table S3.

S3 Extra Results

S3.1 Four multiplets

The result of applying CUPID to the four multiplets datasets is presented in Figure S6, Page S24. These datasets are examples of cases where the direct-dimension is too crowded for reasonable estimates of model order to be made using the MDL, so a value was manually provided; for each dataset, $M = 36$ was set. In all cases except one, the final number of model oscillators was 32, corresponding to the true number of signals in the region considered. For the first example (LHS of Figure S6), the final model order was 33. The position of the extraneous signal is denoted as a purple star in panel d. This signal was removed using the multiplet assignment process, as it violated the first-order signal criteria.

S3.2 Strychnine

The application of CUPID to the simulated strychnine dataset is presented in Figure S8, Page S26. The first-order signals were faithfully quantified, with some signals correspinding to strong coupling artefacts being incorporated into model after optimisation as well. With the execption of a pair of signals located between 7.2 ppm and 7.1 ppm (colured grey in panels c & d), all extraneous signals were removed based on the first-order criteria. The presence of the two grey signals, which correspond to strong coupling artefacts betweeen spins (C) and (D), resulted in a small artefact in the final pure shift spectrum. Since all the coloured multiplets in panel c are derived from only first-order signals, the notable roofing effects present in the conventional 1D spectrum (panel b) are absent. Multiplets corresponding to spins (B), (C), (D), (G) and (H) illustrate this most clearly.

S3.3 Camphor

Figure S9, Page S27 shows the result of applying CUPID to the camphor dataset. The 45°-tilt spectrum (panel a) features peaks with broad lines due to the influence of dispersive character in the 2DJ spectra, as well as numerous additional peaks which arise from strong coupling artefacts. For all but one region, the estimation routine accurately quantified the first order signals, with strong coupling artefacts being either neglected entirely or removed by applying the first order

criteria. In the most shielded region, coupling between spins (F) and (G) yield particularly intense artefacts. Estimated signals corresponding to these are denoted in grey in panels c & d. Inclusion of these in the final pure shift spectrum leads to 3 low-intensity peaks, plotted in grey in panel b (note that these also appear in the 45°-tilt spectrum). It is possible to manually remove these signals; the result is the black spectrum in panel b. This process is analogous to the removal of the water signal in the quinine example (Figure S5, Page S23). Such manual edits to the final result should be clearly documented and rationalised.

S3.4 Dexamethasone

Finally, Figure S10, Page S28 shows the result of applying CUPID to the dexamethasone dataset. For spins (A) and (G), two separate multiplet structures, split only in the direct dimension, could be resolved. These exist due to heteronuclear couplings between these spins and ^{19}F. A number of extraneous signals are present in the final result, which are coloured grey in panels d & e. These are as follows:

- 4.1 ppm: a pair of strong coupling artefacts which lie along the same 45° line.
- 2.1 ppm: A weak signal with an indirect-dimension frequency of 0 Hz. This likely airses from a ^{13}C satellite.
- 1.6 ppm and 1.4 ppm: a pair of weak signals which also have indirect-dimension frequencies of 0 Hz. These correspond to ^{13}C satellites derived from the intense singlet peak associated with the $C(K)_3$ methyl group.

The influence of these signals on the final pure shift spectrum (panel b) is denoted by asterisks. Similar artefacts are also identifiable in the PSYCHE spectrum (panel a).

S4 Figures

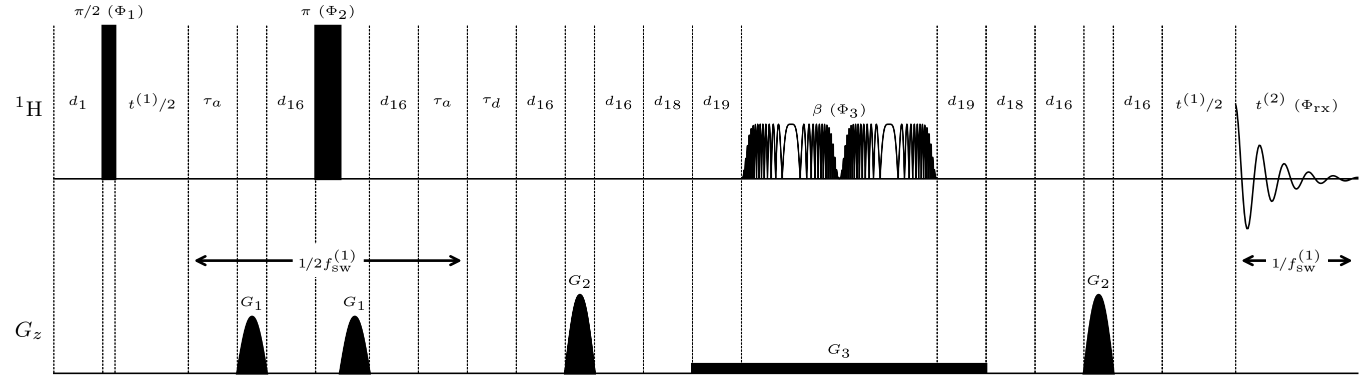

Figure S1: PSYCHE pulse sequence applied to the estradiol sample. All delays are included, though they are not to scale. Delays: d_1 (relaxation delay): 1 s, d_{16}: (gradient recovery delay): 200 µs, d_{18}: 200 µs, d_{19}: 1 ms, τ_a: 1.3 ms, τ_d: 18.9 ms. The PSYCHE element featured two saltire chirp pulses with a WURST amplitude envelope, with a target flip angle $\beta = 20°$. Each saltire pulse had a bandwidth of 10 kHz, a duration of 25 ms, and a power of 280 µW. Hard pulses had a power of 31.537 W, with the duration of the $\pi/2$ pulse being 15 µs. G_1 and G_2 were gradients for coherence order selection. Each comprised a 100-point sine shape profile, and lasted 1 ms. G_3 was a rectangular weak gradient applied during the PSYCHE element, with a duration of 52 ms. The gradeint strengths as a percentage of the maximum permissible z-gradient were, respectively 31%, 47%, 1.6%. The phase cycling scheme used was: $\Phi_1 : 2 \times (0°, 180°)$; $\Phi_2 : 4 \times 0°$; $\Phi_3 : 2 \times 0°, 2 \times 90°$; $\Phi_{rx} : 0°, 180°, 180°, 0°$.

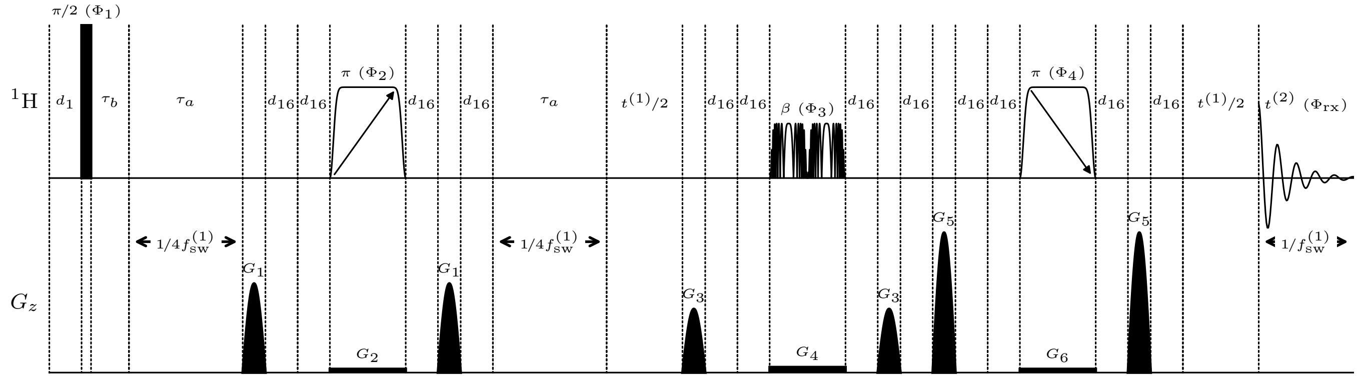

Figure S2: TSE-PSYCHE pulse sequence applied to the dexamethasone sample. Delays: d_1 (relaxation delay): 2 s, d_{16}: (gradient recovery delay): 200 µs, τ_a: 5 ms ($= 1/4f_{sw}^{(1)}$). The hard $\pi/2$ pulse had a duration of 12 µs, and a power of 24 W. The two π pulses were unidirectional frequency-swept (chirped) pulses, with the first pulse sweeping from low to high frequencies, and the second pulse sweeping from high to low. These each had a WURST amplitude envelope, lasted a duration of 40 ms, and had a power of 11.05 mW. The PSYCHE element had a target flip angle $\beta = 15°$, and featured two saltire chirp pulses. Both saltire pulses had a WURST amplitude envelope, a duration of 15 ms, and a power of 1.28 mW. G_1, G_3 and G_5 were gradients for coherence order selection. Each comprised a 100-point sine profile, and lasted 1 ms. G_2, G_4 and G_6 were weak rectangular gradients which were applied at the same time as the chirped pulses. The magnitudes of gradients G_1 to G_6 as a percentage of the maximum permitted gradient were, respectively: 49%, 2%, 35%, 3%, 77%, 2%. The phase cycling scheme used was: $\Phi_1 : 8 \times 0°$; $\Phi_2 : 2 \times (2 \times 0°, 2 \times 180°)$; $\Phi_3 : 2 \times (0°, 90°), 2 \times (180°, 270°)$; $\Phi_4 : 8 \times 0°$; $\Phi_{rx} : 4 \times (0°, 180°)$.

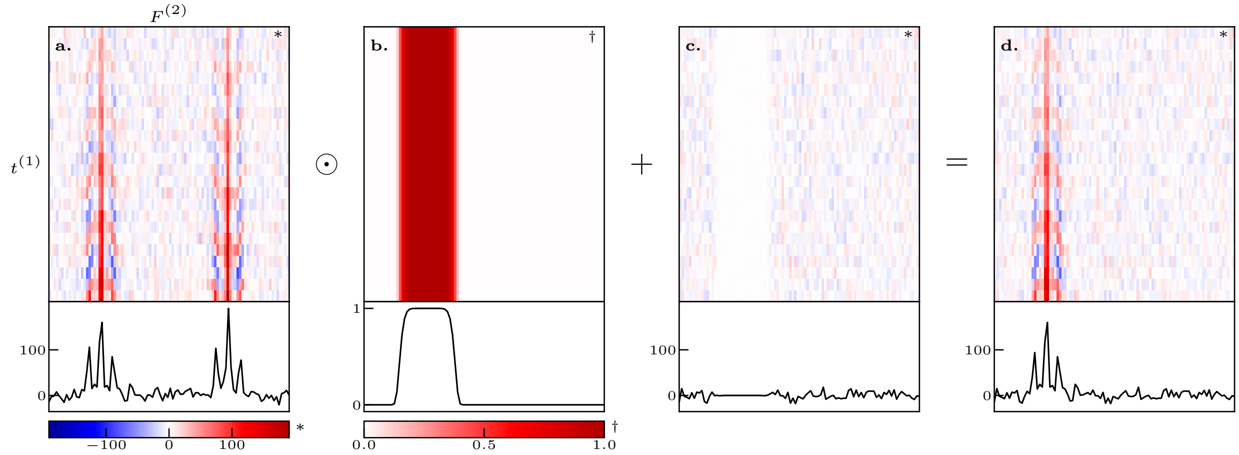

Figure S3: An overview of the frequency-filtration method applied to J-Resolved data. For each panel (a-d), a heat map denotes the entire 2D array, while underneath is a plot of the first slice in the direct dimension. **a.** $\hat{\boldsymbol{S}}$, generated via construction of a virtual echo followed by FT in the direct dimension. **b.** $\boldsymbol{G}$, the super-Gaussian filter used for extracting the spectral region of interest. **c.** $\boldsymbol{W}_{\sigma^2} \odot (\mathbf{1} - \boldsymbol{G})$, a noise array added to ensure regions outside the filter maintain the original noise variance. **d.** $\widetilde{\boldsymbol{S}}$, the resulting filtered spectral data. This subsequently undergoes IFT, and slicing in half in the direct dimension to generate the filtered sub-FID $\widetilde{\boldsymbol{Y}}$.

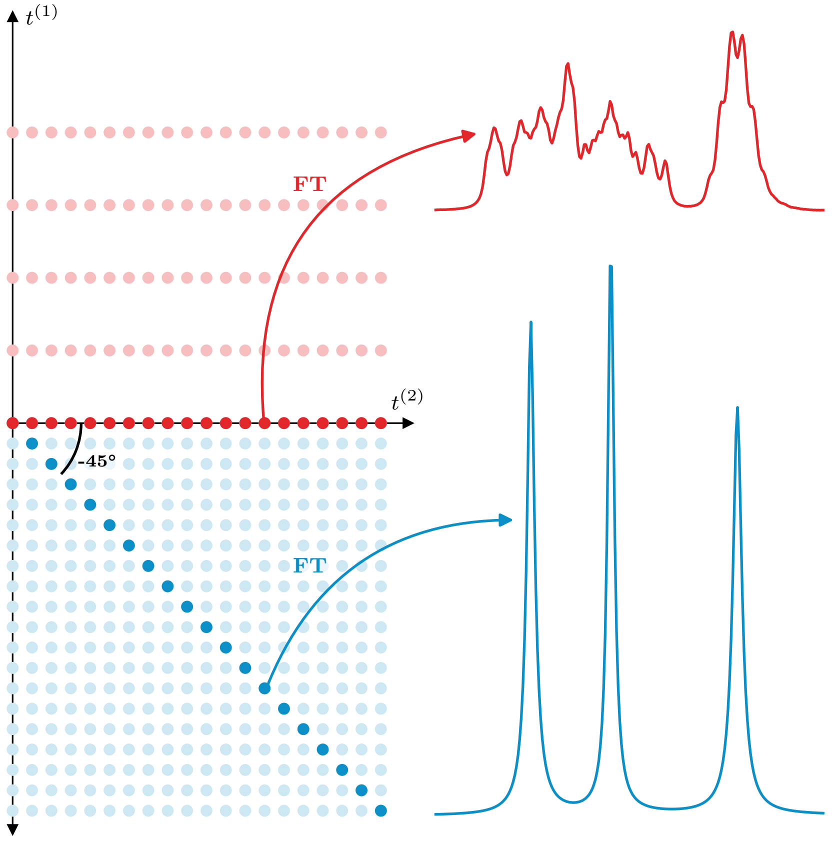

Figure S4: An illustration of the reasoning behind the name "$-45°$ signal". A typical 2DJ FID is denoted in pale red. Note that the rate of sampling in the indirect dimension is far lower relative to the direct dimension ($f_{\text{sw}}^{(1)} \ll f_{\text{sw}}^{(2)}$), and there are far fewer points in the indirect dimension ($N^{(1)} \ll N^{(2)}$). The bright red signal comprises a typical 1D FID, in which contributions from chemical shifts and scalar couplings both feature. The pale blue points denote a hypothetical signal generated by back propagating the 2DJ FID in negative $t^{(1)}$, with the same rate of sampling as in the direct dimension. The main diagonal of this signal, in bright blue, makes a $-45°$ angle with the $t^{(2)}$ axis; the FT of this is a pure-shift spectrum.

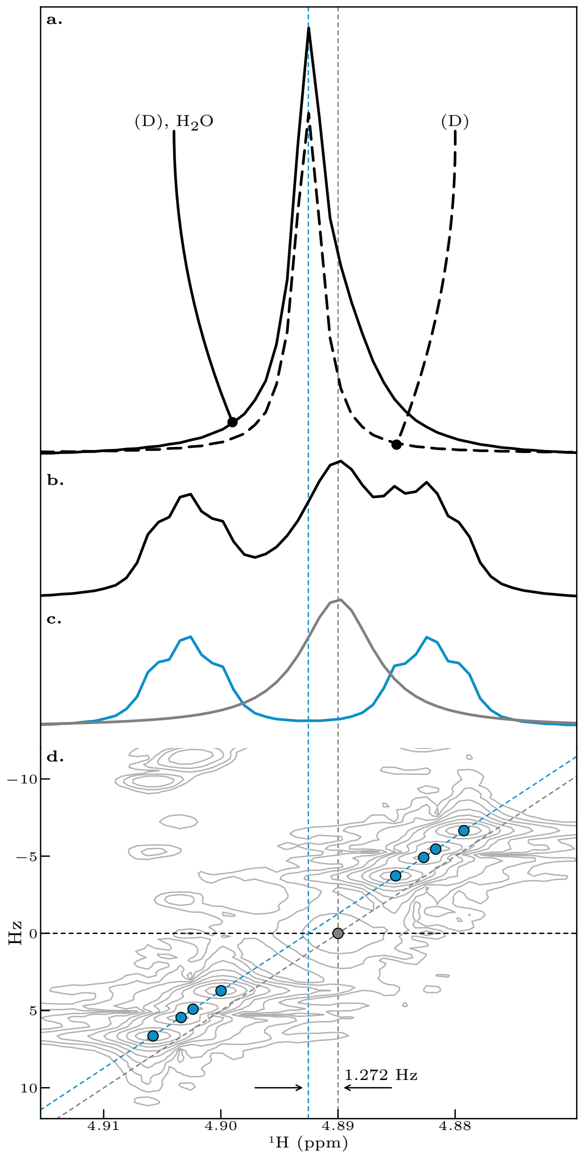

Figure S5: Zoomed-in depiction of the quinine estimation result, focusing on the signals arising from proton (D) and water. **a.** Pure shift spectra generated by CUPID, for cases where the contribution from the water signal was either included (solid) or removed (dashed). **b.** 1D spectrum of the dataset. **c.** Multiplet structures determined with CUPID. **d.** Contour plot of the 2DJ spectrum, with the positions of estimated peaks marked. Dashed vertical blue and grey lines denote the estimated central frequency of the (D) multiplet and the position of the water signal, respectively. Dashed diagonal lines denote the 45° lines along which the multiplet structures lie in the 2DJ spectrum. The (D) multiplet and water signal were determined to be separated by 1.272 Hz. N.B. the threshold used for multiplet prediction was 0.92 Hz.

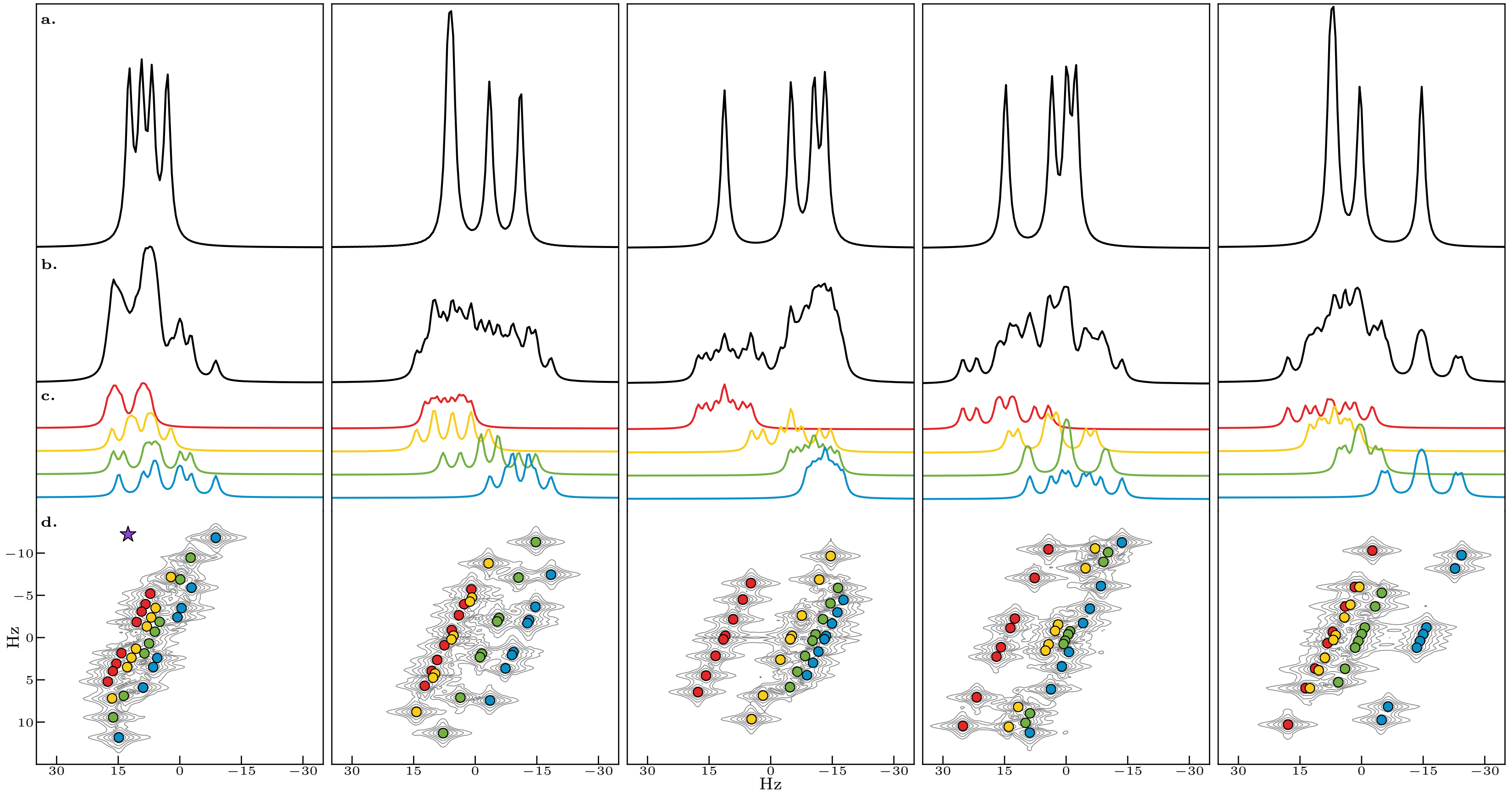

Figure S6: Application of CUPID on 5 simulated 2DJ datasets comprising four heavily overlapping ddd multiplet structures. **a.** Black: pure-shift spectra generated using CUPID. **b.** Spectra produced from the first direct-dimension FIDs in the 2DJ datasets. **c.** Plots of multiplet structures determined ($\epsilon = f_{\mathrm{sw}}^{(2)}/N^{(2)} \approx 0.98\,\mathrm{Hz}$). **d.** Contour plots of the magnitude-mode 2DJ spectra, with coloured points denoting the peak positions of estimated signals. The purple star denotes a signal which was removed using the first-order criteria.

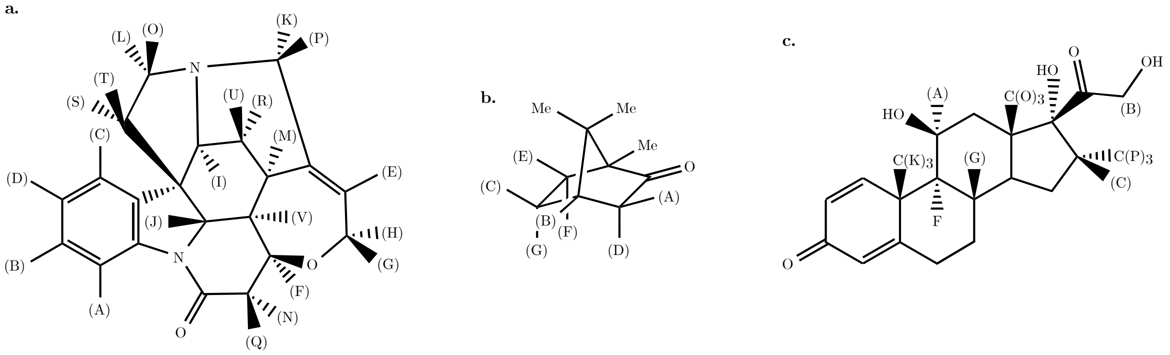

Figure S7: The structures of **a.** strychnine, **b.** camphor, and **c.** dexamethasone.

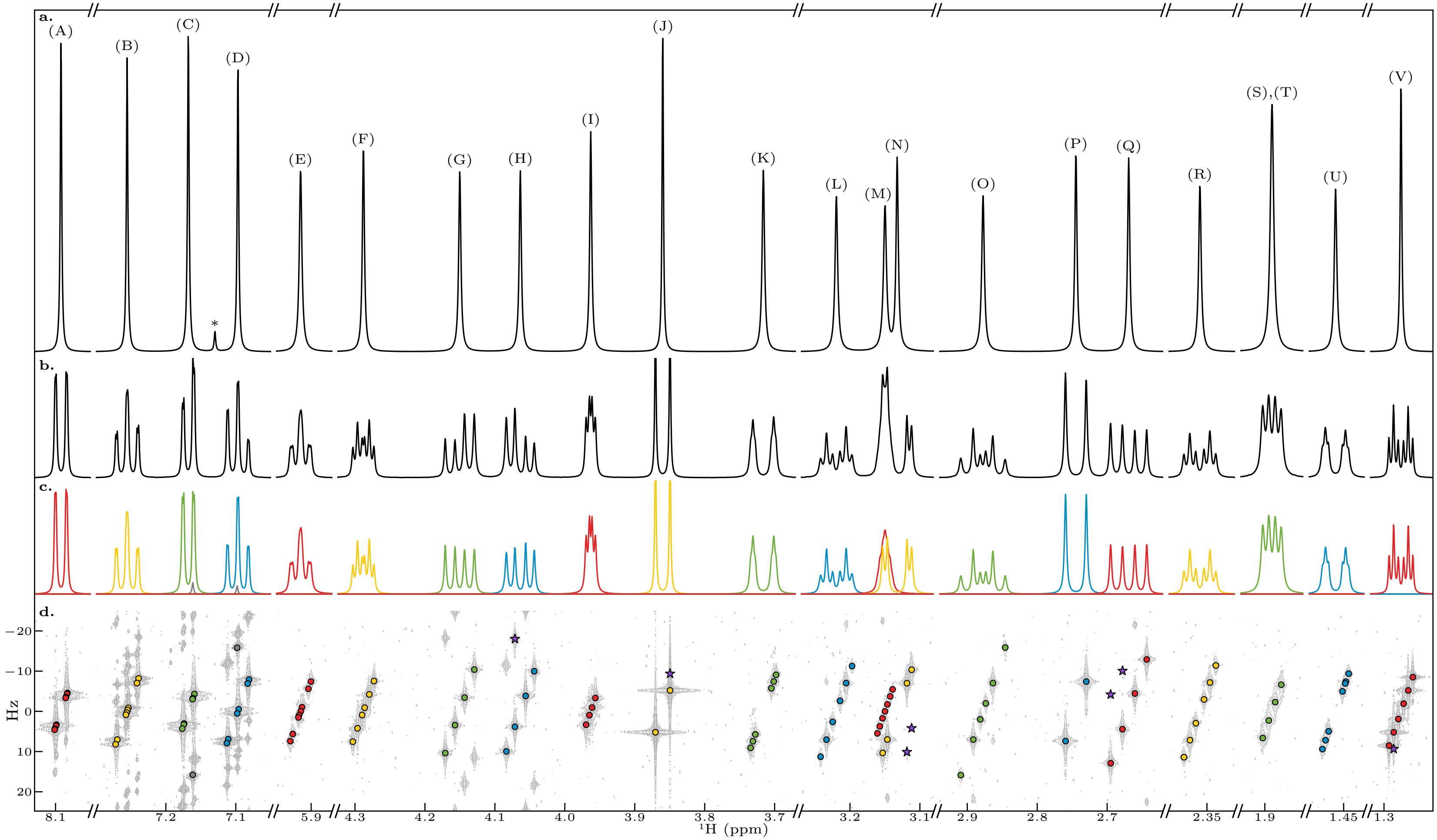

Figure S8: Application of CUPID on the simulated strychnine dataset. **a.** Pure shift spectrum generated. The influence of the estimated strong coupling artefacts is labelled with an asterisk. **b.** Spectrum of the first direct-dimension FID in the 2DJ dataset. **c.** Plots of predicted multiplet structures. **d.** A contour plot of the magnitude-mode 2DJ spectrum. Coloured points locate the frequencies of estimated signals. Purple stars denote estimated signals which were automatically purged based on the first-order criteria, while grey points denote estimated strong coupling artefacts which were not purged.

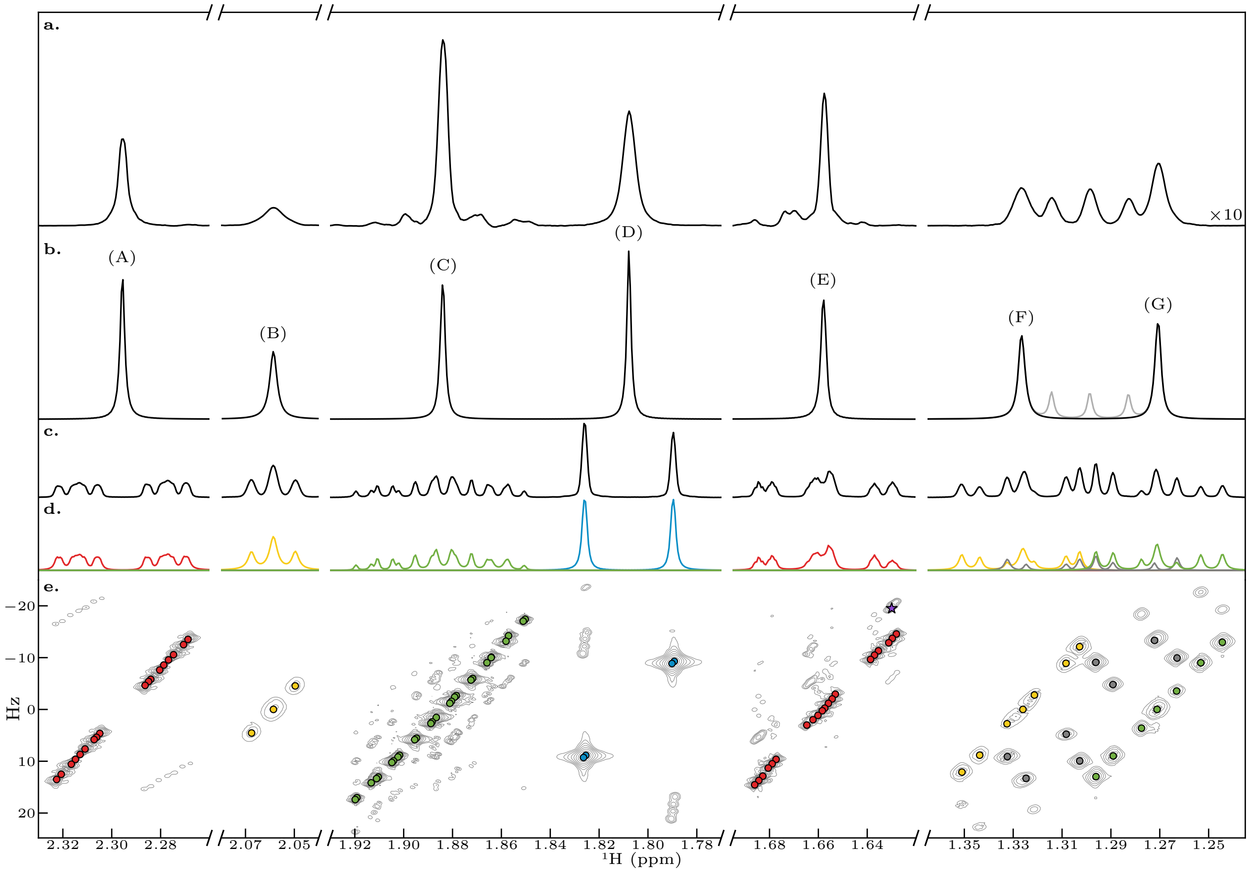

Figure S9: Application of CUPID on the camphor dataset. **a.** Pure shift spectrum generated using the 45°-tilt approach. **b.** Grey: pure shift spectrum generated using CUPID. N.B. outside the 1.33 ppm to 1.27 ppm region, this is indistinguishable from the black spectrum. Black: spectrum after manually neglecting signals related to strong coupling artefacts. **c.** Spectrum produced from the first direct-dimension FID in the 2DJ dataset. **d.** Multiplet structures assigned ($\epsilon = 2f_{\text{sw}}^{(2)}/N^{(2)} \approx 1.23\,\text{Hz}$). **e.** A contour plot of the magnitude-mode 2DJ spectrum. Coloured points denote the positions of estimated signals. Grey points denote those signals which correspond to strong coupling artefacts. The purple star denotes a signal which corresponds to a strong coupling artefact, which was automatically removed based on the first order criteria.

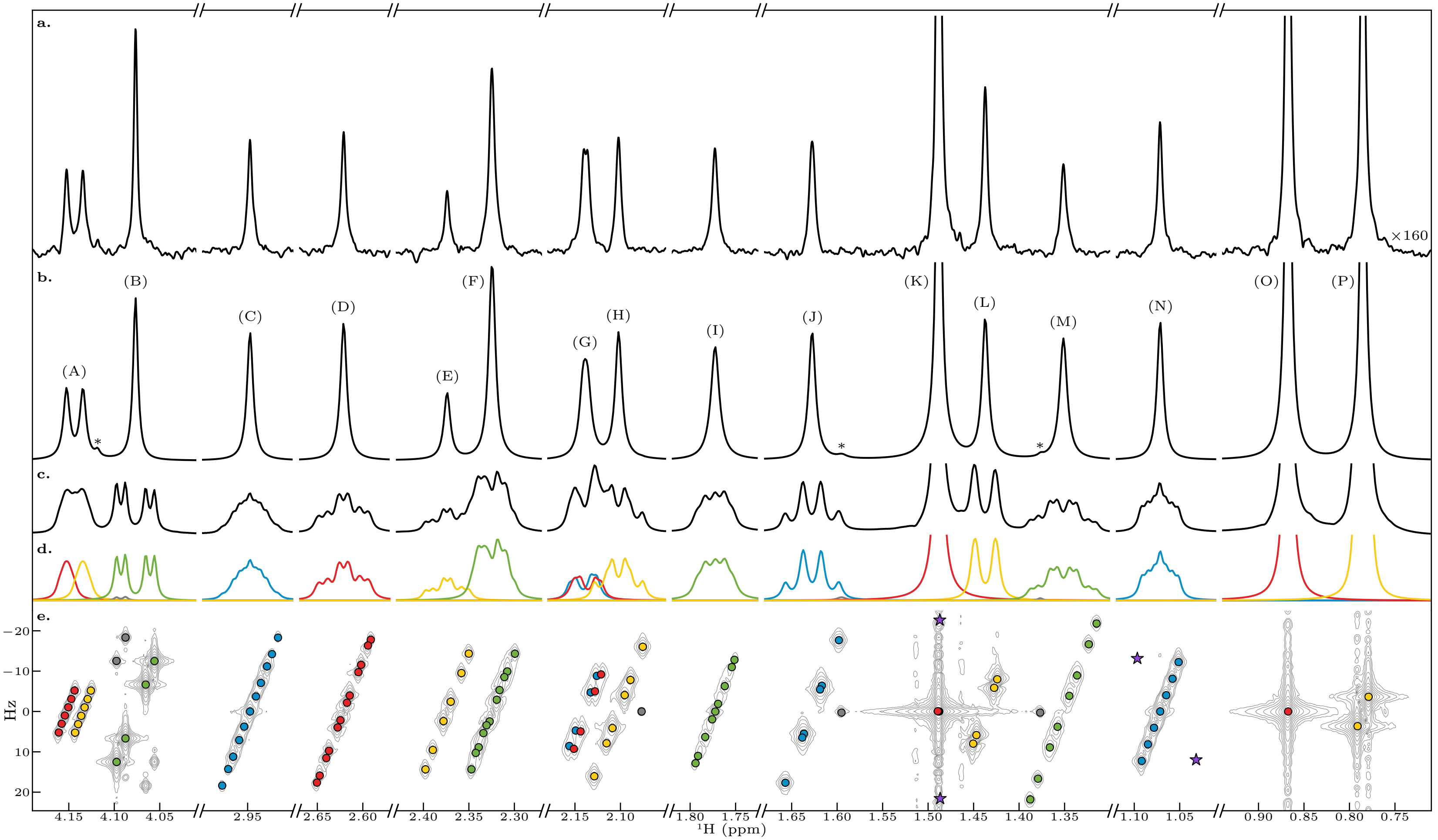

Figure S10: Application of CUPID on dexamethasone dataset. **a.** TSE-PSYCHE spectrum. **b.** Pure shift spectrum generated using CUPID. Asterisks denote features in the spectrum that have been caused by in the incorporation of strong coupling artefacts and ^{13}C satellites. **c.** Spectrum produced from the first direct-dimension FID in the 2DJ dataset. **d.** Multiplet structures assigned ($\epsilon = f_{\mathrm{sw}}^{(1)}/N^{(1)} \approx 1.79\,\mathrm{Hz}$). All the 1D spectra plotted in panels a–d were subjected to 1.12 Hz exponential line-broadening. **e.** A contour plot of the magnitude-mode 2DJ spectrum. Coloured points locate the frequencies of estimated signals in the parameter estimate. Gray points denote estimated signals which do not correspond to first-order multiplet structures; these either derive from strong coupling artefacts (4.1 ppm), or ^{13}C satellites (2.1 ppm, 1.6 ppm and 1.4 ppm). Purple stars denote the positions of estimated signals which were automatically removed using the first-order criteria.

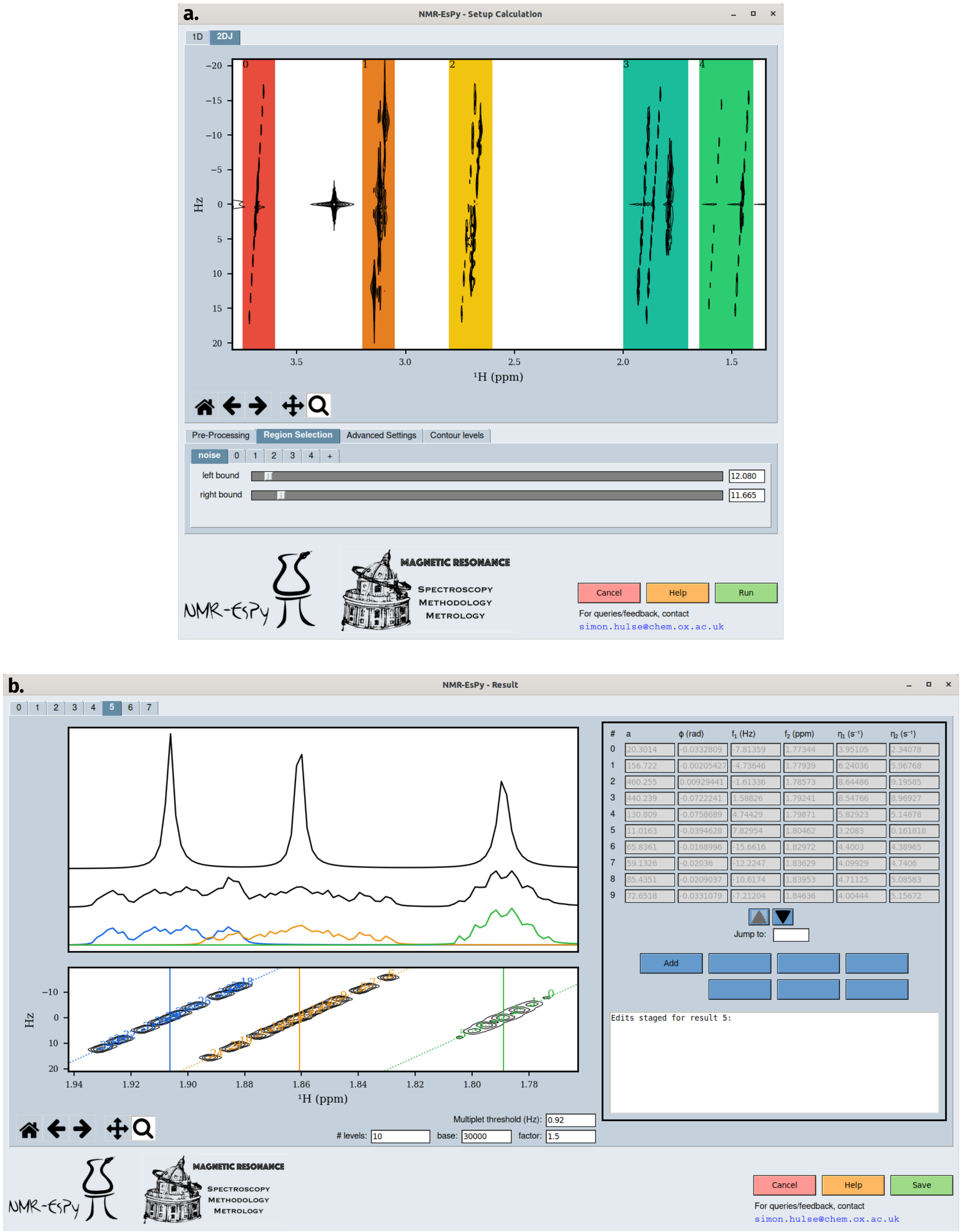

Figure S11: Screenshots of the NMR-EsPy GUI. The exact appearance of the GUI will vary based on the operating system used; these screenshots were obtained on a system running Ubuntu 22.04. **a.** Setup window, used to specify regions of interest (these are highlighted in the 2DJ spectrum), along with other features of the estimation routine. **b.** Result window, where a summary of the result generated by CUPID is provided.

S5 Tables

	Quinine	Estradiol	Camphor	Dexamethasone
SFO1	500.13	500.30	500.13	600.18
O1	2500.0	2501.5	1000.0	2815.4
SW_h[1]	50	100	50	50
SW_h[2]	7500.0	5000.0	5000.0	7211.5
SW[2]	15.00	9.99	10.00	12.02
TD[1]	128	128	128	64
TD[2]	16384	16384	16384	8192
NS	4	4	4	2
DS	4	2	4	8
PLW1	20.893	31.537	20.893	24
P1	10	15	10	12
D1	2	1	2	1.5
TE	298	298	298	298
Time	37:51	28:20	42:33	6:12

Table S1: Noteworthy experiment parameters for the 2DJ and PSYCHE experiments run. The dimension number (`1/2`) is indicated in square brackets for applicable parameters. `SFO1`: Transmitter frequency of ^{1}H channel (MHz); `O1`: Transmitter offset of ^{1}H channel (Hz); `SW/SW_h`: Sweep width (ppm/Hz); `TD`: Number of points acquired (N.B. the number of complex points in the direct-dimension is half of `TD[2]`); `NS`: Number of scans acquired; `DS`: Number of dummy scans; `PLW1`: Hard pulse power (W); `P1`: Duration of 90° pulse (µs); `D1`: Duration of relaxation delay (s); `TE`: Experiment temperature; Time: Estimated experiment time (min), calculated as $N_{\text{incr}} \times t_{\text{avg}}$, where $N_{\text{incr}} = \texttt{DS} + \texttt{NS} \times \texttt{TD[1]}$ and $t_{\text{avg}} = \texttt{D1} + 30\,\text{ms} + 0.5 \times \texttt{TD[1]}/\texttt{SW\_h[1]} + 0.5 \times \texttt{TD[2]}/\texttt{SW\_h[2]}$. All experiments used Bruker type BBO Probes with a nominal maximum z gradient of $53.5\,\text{G}\,\text{cm}^{-1}$. For further experimental details specific to the PSYCHE experiments, see Figures S1 and S2.

Table S2: The isotropic chemical shifts, corresponding resonance frequencies, and scalar couplings associated with spin systems used in SPINACH simulations.

Spin	δ (ppm)	$\omega/2\pi$ (Hz)	J (Hz)
		Four Multiplets - Example 1	
(A)	6.398×10^{-3}	3.199	(E): 5.893, (F): 9.416, (G): 8.343
(B)	1.377×10^{-2}	6.885	(E): 7.567, (F): 2.581, (G): 8.769
(C)	1.880×10^{-2}	9.398	(E): 5.873, (F): 3.697, (G): 4.804
(D)	2.477×10^{-2}	12.383	(E): 1.255, (F): 2.119, (G): 7.036
		Four Multiplets - Example 2	
(A)	-2.189×10^{-2}	-10.947	(E): 5.728, (F): 3.792, (G): 5.372
(B)	-6.665×10^{-3}	-3.332	(E): 9.005, (F): 9.406, (G): 4.220
(C)	1.131×10^{-2}	5.653	(E): 4.041, (F): 4.525, (G): 9.012
(D)	1.329×10^{-2}	6.645	(E): 3.044, (F): 6.609, (G): 1.756
		Four Multiplets - Example 3	
(A)	-2.649×10^{-2}	-13.243	(E): 1.468, (F): 4.641, (G): 2.787
(B)	-2.098×10^{-2}	-10.492	(E): 1.817, (F): 6.223, (G): 3.688
(C)	-9.833×10^{-3}	-4.917	(E): 7.048, (F): 2.796, (G): 9.479
(D)	2.259×10^{-2}	11.294	(E): 4.286, (F): 1.950, (G): 6.662
		Four Multiplets - Example 4	
(A)	-4.486×10^{-3}	-2.243	(E): 9.540, (F): 5.140, (G): 7.820
(B)	-6.200×10^{-6}	-0.003	(E): 9.347, (F): 9.711, (G): 1.132
(C)	7.213×10^{-3}	3.606	(E): 2.339, (F): 9.754, (G): 9.009
(D)	2.971×10^{-2}	14.853	(E): 8.217, (F): 9.312, (G): 3.395
		Four Multiplets - Example 5	
(A)	-2.904×10^{-2}	-14.520	(E): 9.362, (F): 1.595, (G): 8.572
(B)	7.656×10^{-4}	0.383	(E): 1.600, (F): 4.099, (G): 4.873
(C)	1.315×10^{-2}	6.577	(E): 2.121, (F): 3.595, (G): 6.275
(D)	1.548×10^{-2}	7.739	(E): 6.639, (F): 9.632, (G): 4.325
		Strychnine	
(A)	8.092	4044.9	(B): 7.90, (C): 0.23, (D): 0.98
(B)	7.255	3626.5	(A): 7.90, (C): 1.08, (D): 7.44
(C)	7.167	3582.5	(A): 0.23, (B): 1.08, (D): 7.49
(D)	7.098	3548.0	(A): 0.98, (B): 7.44, (C): 7.49
(E)	5.915	2956.7	(G): 7.00, (H): 6.10, (K): 1.79, (M): 0.47
(F)	4.288	2143.4	(N): 3.34, (Q): 8.47, (V): 3.30
(G)	4.148	2073.4	(E): 7.00, (H): -13.80

Continues on next page...

Spin	δ (ppm)	$\omega/2\pi$ (Hz)	J (Hz)
(H)	4.066	2032.4	(E): 6.10, (G): -13.80
(I)	3.963	1981.0	(R): 4.33, (U): 2.42
(J)	3.860	1929.5	(V): 10.41
(K)	3.716	1857.5	(E): 1.79, (M): 1.61, (P): -14.80
(L)	3.219	1609.1	(O): -13.90, (S): 5.50, (T): 3.20
(M)	3.150	1574.6	(E): 0.47, (K): 1.61, (R): 4.11, (U): 1.96, (V): 3.29
(N)	3.132	1565.6	(F): 3.34, (Q): -17.34
(O)	2.878	1438.6	(L): -13.90, (S): 7.20, (T): 10.70
(P)	2.745	1372.1	(K): -14.80
(Q)	2.670	1334.6	(F): 8.47, (N): -17.34
(R)	2.360	1179.7	(I): 4.33, (M): 4.11, (U): -14.35
(S)	1.890	944.7	(L): 5.50, (O): 7.20, (T): -13.90
(T)	1.890	944.7	(L): 3.20, (O): 10.70, (S): -13.90
(U)	1.462	730.8	(I): 2.42, (M): 1.96, (R): -14.35
(V)	1.276	637.8	(F): 3.30, (J): 10.41, (M): 3.29

Table S3: Metrics related to the results generated in this work. *Initial M*: the number of oscillators that were used to construct an initial guess with the MMEMPM. Values marked with an asterisk were manually hard-coded, as opposed to applying the MDL on the first direct-dimension FID. *M after MMEMPM*: the number of oscillators present in $\boldsymbol{\theta}^{(0)}$. This differs from *Initial M* in cases where oscillators possessing negative damping factors were generated by the MMEMPM. *MMEMPM time*: the time taken to generate the initial guess $\boldsymbol{\theta}^{(0)}$. *Opt. iterations*: the number of iterations performed during the optimisation routine. *M after opt.*: the number of oscillators present in $\boldsymbol{\theta}^{(*)}$, which differs from *M after MMEMPM*, in cases where oscillators acquire negative amplitudes during the routine. *Opt. time*: the time taken to perform the optimisation routine. *Spurious oscs*: the number of oscillators removed from $\boldsymbol{\theta}^{(*)}$, based on the first-order signal criteria.

Region (ppm)	Initial M	M after MMEMPM	MMEMPM time (s)	Opt. iterations	M after opt.	Opt. time (s)	Spurious oscs.
			Quinine				
5.8 – 5.55	18	18	6.67	154	10	4.64	0
5.0 – 4.85	17*	17	3.94	15	17	0.55	0
3.75 – 3.63	14	14	3.10	109	13	1.81	0
3.17 – 3.06	15	15	4.55	182	11	2.31	0
2.8 – 2.6	20	20	4.74	161	18	5.28	0
2.0 – 1.7	36*	36	10.86	145	33	14.14	0
1.64 – 1.52	16	16	3.59	192	15	4.39	0
1.52 – 1.4	18	18	6.72	528	16	9.39	2
			Estradiol				
2.29 – 2.17	20*	18	19.09	125	12	2.99	1
2.12 – 2.0	15*	15	16.52	78	7	1.48	0
1.95 – 1.72	40*	40	29.95	125	33	18.57	1
1.65 – 1.52	20*	19	15.77	125	16	4.13	0
1.45 – 1.02	90*	89	31.47	150	84	92.21	3
			Four Multiplets				
Example 1	36*	36	8.93	270	34	11.97	2
Example 2	36*	36	9.16	33	32	1.65	0

Continues on next page...

Region (ppm)	Initial M	M after MMEMPM	MMEMPM time (s)	Opt. iterations	M after opt.	Opt. time (s)	Spurious oscs.
Example 3	36^*	36	8.76	41	32	2.07	0
Example 4	36^*	36	8.05	31	32	1.55	0
Example 5	36^*	36	8.60	46	32	2.28	0
			Strychnine				
8.14 – 8.05	8	8	2.02	19	8	0.29	0
7.3 – 7.05	22	22	8.25	44	22	3.75	0
5.96 – 5.87	8	8	2.44	80	8	0.76	0
4.35 – 4.0	15	15	6.13	10	15	1.12	1
4.0 – 3.64	13	13	6.47	18	13	1.70	1
3.3 – 3.05	20^*	20	6.39	44	19	3.58	2
3.0 – 2.56	15	15	6.32	24	14	2.69	2
2.45 – 2.25	7	7	4.25	16	6	0.44	0
2.0 – 1.74	5	5	5.79	31	4	0.60	0
1.55 – 1.35	7	7	3.55	41	7	0.86	0
1.35 – 1.2	8	8	3.44	24	7	0.50	1
			Camphor				
2.35 – 2.23	18	18	5.46	82	18	2.37	0
2.09 – 2.025	7	7	3.03	47	3	0.32	0
1.95 – 1.75	35	32	11.15	550	31	45.60	0
1.7 – 1.61	21	21	6.28	72	21	2.75	1
1.375 – 1.215	29	29	6.86	102	22	5.33	0
			Dexamethasone				
7.45 – 7.15	8	8	3.29	59	2	0.35	0
6.4 – 5.9	15	15	6.80	225	8	2.56	1

Continues on next page...

Region (ppm)	Initial M	M after MMEMPM	MMEMPM time (s)	Opt. iterations	M after opt.	Opt. time (s)	Spurious oscs.
5.5 – 4.8	20	19	8.08	268	6	4.37	1
4.8 – 4.3	11	11	4.13	50	9	0.72	2
4.25 – 3.97	22*	20	5.43	48	18	1.05	0
3.0 – 2.87	13	11	3.46	18	11	0.22	0
2.68 – 2.565	13	12	3.52	54	12	0.41	0
2.413 – 2.26	17	17	4.04	28	17	0.49	0
2.2 – 2.03	16	16	4.37	75	15	1.28	0
1.85 – 1.7	12	11	7.07	48	9	0.32	0
1.7 – 1.25	29	29	7.11	250	25	8.41	2
1.14 – 1.0	12	10	5.41	110	9	0.62	2
1.0 – 0.65	3*	3	1.79	9	3	0.14	0

S6 Algorithms

Algorithm S1 The numerical optimisation routine employed in NMR-EsPy, and used for all results generated in this work. This makes use of Algorithms 4.1 & 7.2 in [3], with an extra check inserted to deal with any negative-amplitude oscillators which may be generated as the routine evolves (lines 19 – 22).

1: **procedure** NLP($\boldsymbol{Y}$, $\boldsymbol{\theta}^{(0)}$)
2: $\quad\Delta^{(0)} \leftarrow {}^{1}/_{10}\, \|\nabla\mathcal{F}(\boldsymbol{\theta}^{(k)}|\boldsymbol{Y})\|$; *Initial trust region radius*
3: $\quad\Delta_{\text{max}} \leftarrow 16\Delta^{(0)}$; *Maximum permitted trust region radius*
4: $\quad$**for** $k = \{0, 1, \cdots\}$ **do**
5: $\quad\quad\boldsymbol{p}^{(k)} \leftarrow \textsc{SteihaugToint}\,(\boldsymbol{Y}, \boldsymbol{\theta}^{(k)}, \Delta^{(k)})$; *Algorithm S2*
6: $\quad\quad\rho^{(k)} \leftarrow \dfrac{\mathcal{F}(\boldsymbol{\theta}^{(k)}|\boldsymbol{Y}) - \mathcal{F}(\boldsymbol{\theta}^{(k)} + \boldsymbol{p}^{(k)}|\boldsymbol{Y})}{\mathcal{F}_{\text{Q}}(\boldsymbol{\theta}^{(k)}|\boldsymbol{Y}) - \mathcal{F}_{\text{Q}}(\boldsymbol{\theta}^{(k)} + \boldsymbol{p}^{(k)}|\boldsymbol{Y})}$; *Measure of efficacy of quadratic approximation (QA)*
7: $\quad\quad$**if** $\rho^{(k)} < {}^{1}/_{4}$ **then**
8: $\quad\quad\quad\Delta^{(k+1)} \leftarrow {}^{1}/_{4}\Delta^{(k)}$; *QA performing badly, reduce trust region radius*
9: $\quad\quad$**else if** $\rho^{(k)} > {}^{3}/_{4}$ **and** $\|\boldsymbol{p}^{(k)}\| = \Delta^{(k)}$ **then**
10: $\quad\quad\quad\Delta^{(k+1)} \leftarrow \min\,(2\Delta^{(k)}, \Delta_{\text{max}})$; *QA performing well, increase trust region radius*
11: $\quad\quad$**else**
12: $\quad\quad\quad\Delta^{(k+1)} \leftarrow \Delta^{(k)}$;
13: $\quad\quad$**end if**
14: $\quad\quad$**if** $\rho^{(k)} > {}^{3}/_{20}$ **then**
15: $\quad\quad\quad\boldsymbol{\theta}^{(k+1)} \leftarrow \boldsymbol{\theta}^{(k)} + \boldsymbol{p}^{(k)}$; *QA performing sufficiently well to update parameter vector*
16: $\quad\quad$**else**
17: $\quad\quad\quad\boldsymbol{\theta}^{(k+1)} \leftarrow \boldsymbol{\theta}^{(k)}$; *QA performing too poorly to justify updating parameter vector*
18: $\quad\quad$**end if**
19: $\quad\quad$**if** $k \bmod 25 = 0$ **and** $\boldsymbol{\theta}^{(k+1)}$ contains negative amplitudes **then**
20: $\quad\quad\quad\boldsymbol{\theta}^{(0)} \leftarrow \boldsymbol{\theta}^{(k+1)}$ with negative amplitudes removed;
21: $\quad\quad\quad\boldsymbol{\theta}^{(*)} \leftarrow \text{NLP}\,(\boldsymbol{Y}, \boldsymbol{\theta}^{(0)})$;
22: $\quad\quad$**end if**
23: $\quad\quad$**if** $\|\nabla\mathcal{F}(\boldsymbol{\theta}^{(k+1)}|\boldsymbol{Y})\| < 10^{-8}$ **then**
24: $\quad\quad\quad\boldsymbol{\theta}^{(*)} \leftarrow \boldsymbol{\theta}^{(k+1)}$;
25: $\quad\quad\quad$**break**; *Convergence achieved; break from loop*
26: $\quad\quad$**end if**
27: $\quad$**end for**
28: $\quad$**return** $\boldsymbol{\theta}^{(*)}$;
29: **end procedure**

Algorithm S2 The Steihaug-Toint method for computing an appropriate update of the parameter guess during numerical optimisation. This algorithm is equivalent to Algorithm 7.2 in [3].

procedure SteihaugToint($\boldsymbol{Y}$, $\boldsymbol{\theta}^{(k)}$, $\mathit{\Delta}^{(k)}$)
  $\boldsymbol{g} \leftarrow \nabla \mathcal{F}(\boldsymbol{\theta}^{(k)}|\boldsymbol{Y})$; *Equation 3b*
  $\boldsymbol{H} \leftarrow \nabla^2 \mathcal{F}(\boldsymbol{\theta}^{(k)}|\boldsymbol{Y})$; *Equation 9*
  $\epsilon^{(k)} \leftarrow \min\left(1/2, \sqrt{\|\boldsymbol{g}\|}\right)\|\boldsymbol{g}\|$;
  $\boldsymbol{z}^{(0)} \leftarrow \mathbf{0} \in \mathbb{R}^{6M}$;
  $\boldsymbol{r}^{(0)} \leftarrow \boldsymbol{g}$;
  $\boldsymbol{d}^{(0)} \leftarrow -\boldsymbol{r}^{(0)}$;
  if $\|\boldsymbol{r}^{(0)}\| < \epsilon^{(k)}$ **then**
    return $\boldsymbol{z}^{(0)}$;
  end if
  for $j = 0, 1, \cdots$ **do**
    if $\boldsymbol{d}^{(j)\mathrm{T}}\boldsymbol{H}\boldsymbol{d}^{(j)} \leq 0$ **then**
      Find τ such that $\boldsymbol{p}^{(k)} = \boldsymbol{z}^{(k)} + \tau\boldsymbol{d}^{(k)}$ minimises $\mathcal{F}_{\mathrm{Q}}(\boldsymbol{\theta}^{(k)} + \boldsymbol{p}^{(k)}|\boldsymbol{Y})$, s.t. $\|\boldsymbol{p}^{(k)}\| = \mathit{\Delta}^{(k)}$;
      return $\boldsymbol{p}^{(k)}$;
    end if
    $\alpha^{(j)} \leftarrow \dfrac{\boldsymbol{r}^{(j)\mathrm{T}}\boldsymbol{r}^{(j)}}{\boldsymbol{d}^{(j)\mathrm{T}}\boldsymbol{H}\boldsymbol{d}^{(j)}}$
    $\boldsymbol{z}^{(j+1)} \leftarrow \boldsymbol{z}^{(j)} + \alpha^{(j)}\boldsymbol{d}^{(j)}$;
    if $\|\boldsymbol{z}^{(j+1)}\| < \epsilon^{(k)}$ **then**
      Find $\tau \in \mathbb{R}_{>0}$ such that $\boldsymbol{p}^{(k)} = \boldsymbol{z}^{(j)} + \tau\boldsymbol{d}^{(j)}$ satisfies $\|\boldsymbol{p}^{(k)}\| = \mathit{\Delta}^{(k)}$;
      return $\boldsymbol{p}^{(k)}$;
    end if
    $\boldsymbol{r}^{(j+1)} \leftarrow \boldsymbol{r}^{(j)} + \alpha^{(j)}\boldsymbol{H}\boldsymbol{d}^{(j)}$;
    if $\|\boldsymbol{r}^{(j+1)}\| < \epsilon^{(k)}$ **then**
      return $\boldsymbol{z}^{(j+1)}$;
    end if
    $\beta^{(j+1)} \leftarrow \dfrac{\boldsymbol{r}^{(j+1)\mathrm{T}}\boldsymbol{r}^{(j+1)}}{\boldsymbol{r}^{(j)\mathrm{T}}\boldsymbol{r}^{(j)}}$;
    $\boldsymbol{d}^{(j+1)} \leftarrow -\boldsymbol{r}^{(j+1)} + \beta^{(j+1)}\boldsymbol{d}^{(j)}$;
  end for
end procedure

Algorithm S3 The MMEMPM. TruncatedSVD($\boldsymbol{A}, n$) returns the n most significant left singular vectors (matrix $\boldsymbol{U}$), singular values (vector $\boldsymbol{\sigma}$), and right singular vectors (matrix $\boldsymbol{V}^\dagger$) of matrix $\boldsymbol{A}$. Eigendecomposition($\boldsymbol{A}$) returns the eigenvalues and eigenvectors of matrix $\boldsymbol{A}$. Eigenvalues($\boldsymbol{A}$) returns the eigenvalues of matrix $\boldsymbol{A}$. $\boldsymbol{e}(i)$ denotes a vector of length $L^{(1)}L^{(2)}$ where every element is 0 except the i-th which is 1. N.B. Any two frequencies $f_i^{(1)}, f_j^{(1)} \in \boldsymbol{f}^{(1)}$ are deemed to be distinct if $|f_i^{(1)} - f_j^{(1)}| > f_{\text{sw}}^{(1)}/N^{(1)}$.

procedure MMEMPM($\boldsymbol{Y}$, M)

$L^{(1)} \leftarrow \lfloor N^{(1)}/2 \rfloor$;

$L^{(2)} \leftarrow \lfloor N^{(2)}/2 \rfloor$;

for $n^{(1)} = 1, \cdots, N^{(1)}$ **do**

$$\boldsymbol{H}_{n^{(1)}} \leftarrow \begin{bmatrix} y_{n^{(1)},1} & y_{n^{(1)},2} & \cdots & y_{n^{(1)},N^{(2)}-L^{(2)}+1} \\ y_{n^{(1)},2} & y_{n^{(1)},3} & \cdots & y_{n^{(1)},N^{(2)}-L^{(2)}+2} \\ \vdots & \vdots & \ddots & \vdots \\ y_{n^{(1)},L^{(2)}} & y_{n^{(1)},L^{(2)}+1} & \cdots & y_{n^{(1)},N^{(2)}} \end{bmatrix}$$

Hankel matrix of points in $n^{(1)}$-th direct-dimension FID

end for;

$$\boldsymbol{H}_e \leftarrow \begin{bmatrix} \boldsymbol{H}_1 & \boldsymbol{H}_2 & \cdots & \boldsymbol{H}_{N^{(1)}-L^{(1)}+1} \\ \boldsymbol{H}_2 & \boldsymbol{H}_3 & \cdots & \boldsymbol{H}_{N^{(1)}-L^{(1)}+2} \\ \vdots & \vdots & \ddots & \vdots \\ \boldsymbol{H}_{L^{(1)}} & \boldsymbol{H}_{L^{(1)}+1} & \cdots & \boldsymbol{H}_{N^{(1)}} \end{bmatrix};$$

Enhanced Hankel matrix

$\boldsymbol{U}, \boldsymbol{\sigma}, \boldsymbol{V}^\dagger \leftarrow$ TruncatedSVD $(\boldsymbol{H}_e, M)$;

$\boldsymbol{U}_1 \leftarrow \boldsymbol{U}$ with last $L^{(2)}$ rows removed;

$\boldsymbol{U}_2 \leftarrow \boldsymbol{U}$ with first $L^{(2)}$ rows removed;

$\boldsymbol{z}^{(1)}, \boldsymbol{W}^{(1)} \leftarrow$ Eigendecomposition $\left(\boldsymbol{U}_1^+ \boldsymbol{U}_2\right)$; *$\boldsymbol{z}^{(1)}$: signal poles of indirect dimension*

$\boldsymbol{f}^{(1)} \leftarrow (f_{\text{sw}}^{(1)}/2\pi) \operatorname{Im}(\ln \boldsymbol{z}^{(1)})$;

$\boldsymbol{\eta}^{(1)} \leftarrow -f_{\text{sw}}^{(1)} \operatorname{Re}(\ln \boldsymbol{z}^{(1)})$;

Continues on next page...

$$\boldsymbol{P} \leftarrow \begin{bmatrix} \boldsymbol{e}(1)^{\mathrm{T}} \\ \boldsymbol{e}(L^{(2)}+1)^{\mathrm{T}} \\ \vdots \\ \boldsymbol{e}((L^{(1)}-1)L^{(2)}+1)^{\mathrm{T}} \\ \boldsymbol{e}(2)^{\mathrm{T}} \\ \boldsymbol{e}(L^{(2)}+2)^{\mathrm{T}} \\ \vdots \\ \boldsymbol{e}((L^{(1)}-1)L^{(2)}+2)^{\mathrm{T}} \\ \vdots \\ \vdots \\ \boldsymbol{e}(L^{(2)})^{\mathrm{T}} \\ \boldsymbol{e}(2L^{(2)})^{\mathrm{T}} \\ \vdots \\ \boldsymbol{e}(L^{(1)}L^{(2)})^{\mathrm{T}} \end{bmatrix};$$ *Permutation Matrix*

$\boldsymbol{U}_{\mathrm{P}} \leftarrow \boldsymbol{PU}$

$\boldsymbol{U}_{\mathrm{P1}} \leftarrow \boldsymbol{U}_{\mathrm{P}}$ with last $L^{(1)}$ rows removed;

$\boldsymbol{U}_{\mathrm{P2}} \leftarrow \boldsymbol{U}_{\mathrm{P}}$ with first $L^{(1)}$ rows removed;

$\boldsymbol{G} \leftarrow [\boldsymbol{W}^{(1)}]^{-1}\, \boldsymbol{U}_{\mathrm{P1}}^{+} \boldsymbol{U}_{\mathrm{P2}} \boldsymbol{W}^{(1)}$;

if all values in $\boldsymbol{f}^{(1)}$ are distinct **then**

  $\boldsymbol{z}^{(2)} \leftarrow \mathrm{diag}(\boldsymbol{G})$;

else

  $\boldsymbol{z}^{(2)} \leftarrow \boldsymbol{0} \in \mathbb{C}^{M}$

  $R \leftarrow$ number unique frequencies in $\boldsymbol{f}^{(1)}$; *N.B.* $R < M$

  $\mathbb{R}^{R} \ni \boldsymbol{\chi}^{(1)} \leftarrow$ set of unique frequencies in $\boldsymbol{f}^{(1)}$;

  for $r = 1, \cdots, R$ **do**

    $\boldsymbol{\mu}_r \leftarrow$ positions in $\boldsymbol{f}^{(1)}$ which match with $\chi_r^{(1)}$;

    $h_r \leftarrow$ number of elements in $\boldsymbol{\mu}_r$;

    $\mathbb{R}^{h_r \times h_r} \ni \boldsymbol{G}_r \leftarrow$ sub-matrix of $\boldsymbol{G}$ comprising the rows and columns given by $\boldsymbol{\mu}_r$;

    $\boldsymbol{z}_r^{(2)} \leftarrow$ EIGENVALUES($\boldsymbol{G}_r$);

    assign values of $\boldsymbol{z}_r^{(2)}$ to $\boldsymbol{z}^{(2)}$, according to indices $\boldsymbol{\mu}_r$;

  end for;

end if;

$\boldsymbol{f}^{(2)} \leftarrow (f_{\mathrm{sw}}^{(2)}/2\pi)\, \mathrm{Im}\,(\ln \boldsymbol{z}^{(2)}) + f_{\mathrm{off}}$;

$\boldsymbol{\eta}^{(2)} \leftarrow -f_{\mathrm{sw}}^{(2)}\, \mathrm{Re}\,(\ln \boldsymbol{z}^{(2)})$;

Continues on next page...

$$\boldsymbol{L}^{(2)} \leftarrow \begin{bmatrix} 1 & 1 & \cdots & 1 \\ z_1^{(2)} & z_2^{(2)} & \cdots & z_M^{(2)} \\ \left[z_1^{(2)}\right]^2 & \left[z_2^{(2)}\right]^2 & \cdots & \left[z_M^{(2)}\right]^2 \\ \vdots & \vdots & \ddots & \vdots \\ \left[z_1^{(2)}\right]^{L^{(2)}-1} & \left[z_2^{(2)}\right]^{L^{(2)}-1} & \cdots & \left[z_M^{(2)}\right]^{L^{(2)}-1} \end{bmatrix};$$

$$\boldsymbol{R}^{(2)} \leftarrow \begin{bmatrix} 1 & z_1^{(2)} & \left[z_1^{(2)}\right]^2 & \cdots & \left[z_1^{(2)}\right]^{N^{(2)}-L^{(2)}} \\ 1 & z_2^{(2)} & \left[z_2^{(2)}\right]^2 & \cdots & \left[z_2^{(2)}\right]^{N^{(2)}-L^{(2)}} \\ \vdots & \vdots & \vdots & \ddots & \vdots \\ 1 & z_M^{(2)} & \left[z_M^{(2)}\right]^2 & \cdots & \left[z_M^{(2)}\right]^{N^{(2)}-L^{(2)}} \end{bmatrix};$$

$$\boldsymbol{D}^{(1)} \leftarrow \begin{bmatrix} z_1^{(1)} & 0 & \cdots & 0 \\ 0 & z_2^{(1)} & \cdots & 0 \\ \vdots & \vdots & \ddots & \vdots \\ 0 & 0 & \cdots & z_M^{(1)} \end{bmatrix};$$

$$\boldsymbol{L}_e \leftarrow \begin{bmatrix} \boldsymbol{L}^{(2)} \\ \boldsymbol{L}^{(2)}\boldsymbol{D}^{(1)} \\ \vdots \\ \boldsymbol{L}^{(2)}\left[\boldsymbol{D}^{(1)}\right]^{K-1} \end{bmatrix};$$

$$\boldsymbol{R}_e \leftarrow \left[\boldsymbol{R}^{(2)} \;\; \boldsymbol{D}^{(1)}\boldsymbol{L}^{(2)} \;\; \cdots \;\; \left[\boldsymbol{D}^{(1)}\right]^{N^{(1)}-K}\boldsymbol{L}^{(2)}\right];$$

$\boldsymbol{\alpha} \leftarrow \operatorname{diag}\left(\boldsymbol{L}_e^{+}\boldsymbol{H}_e\boldsymbol{R}_e^{+}\right);$

$\boldsymbol{a} \leftarrow |\boldsymbol{\alpha}|$

$\boldsymbol{\phi} \leftarrow \operatorname{arctan2}\left(\operatorname{Im}(\boldsymbol{\alpha}), \operatorname{Re}(\boldsymbol{\alpha})\right)$

$\boldsymbol{\theta}^{(0)} \leftarrow \left[\boldsymbol{a} \;\; \boldsymbol{\phi} \;\; \boldsymbol{f}^{(1)} \;\; \boldsymbol{f}^{(2)} \;\; \boldsymbol{\eta}^{(1)} \;\; \boldsymbol{\eta}^{(2)}\right]^{\mathrm{T}};$

return $\boldsymbol{\theta}^{(0)}$

end procedure

Algorithm S4 The MDL for approximating the model order M of a 2DJ FID.

procedure MDL($\boldsymbol{Y} \in \mathbb{C}^{N^{(1)} \times N^{(2)}}$)

$L^{(2)} \leftarrow \lfloor N^{(2)}/3 \rfloor$; *Pencil parameter*

$$\boldsymbol{H}_1 \leftarrow \begin{bmatrix} y_{1,1} & y_{1,2} & \cdots & y_{1,L^{(2)}+1} \\ y_{1,2} & y_{1,3} & \cdots & y_{1,L^{(2)}+2} \\ \vdots & \vdots & \ddots & \vdots \\ y_{1,N^{(2)}-L^{(2)}} & y_{1,N^{(2)}-L^{(2)}+1} & \cdots & y_{1,N^{(2)}} \end{bmatrix};$$ *Hankel matrix of first direct-dimension FID*

$\boldsymbol{U}, \boldsymbol{\sigma}, \boldsymbol{V}^\dagger \leftarrow \mathrm{SVD}\left(\boldsymbol{H}_1\right)$;

$\nu_{\min} \leftarrow \infty$;

$M \leftarrow -1$;

for $k = 0, 1, \cdots, L^{(2)} - 1$ **do**

$$\nu \leftarrow -\ln\left(\frac{\prod_{i=k+1}^{L^{(2)}} \sigma_i^{1/(L^{(2)}-k)}}{1/(L^{(2)}-k)\sum_{i=k+1}^{L^{(2)}} \sigma_i}\right)^{(L^{(2)}-k)N^{(2)}} + \frac{1}{2}k(2L^{(2)}-k)\ln N^{(2)};$$

if $\nu < \nu_{\min}$ **then**

$\nu_{\min} \leftarrow \nu$;

$M \leftarrow k$;

end if

end for;

return M;

end procedure

Algorithm S5 Frequency-filtration procedure. RANDOMGAUSSIAN(μ, σ^2, m, n) generates a matrix of shape $m \times n$, where each element has been randomly sampled from a Normal (Gaussian) distribution with mean μ and variance σ^2. $\boldsymbol{r}$ comprises the indices of the left and right bounds of the region of interest, while $\boldsymbol{n}$ comprises the analogous bounds for the noise region. Typically, these will be provided in units of ppm by the user; conversion to array indices is achieved through Equation 13, Page S13.

procedure FILTER($\boldsymbol{Y} \in \mathbb{C}^{N^{(1)} \times N^{(2)}}$, $\boldsymbol{r} \in [[1, 2N^{(2)}]]^2$, $\boldsymbol{n} \in [[1, 2N^{(2)}]]^2$, $\gamma \in \mathbb{R}_{>1}$)

$$\hat{\boldsymbol{Y}} \leftarrow \begin{bmatrix} \mathrm{Re}(y_{1,1}) & y_{1,2} & \cdots & y_{1,N^{(2)}} & 0 & y^*_{1,N^{(2)}} & y^*_{1,N^{(2)}-1} & y^*_{1,2} \\ \vdots & \vdots & \vdots & \vdots & \vdots & \vdots & \vdots & \vdots \\ \mathrm{Re}(y_{N^{(1)},1}) & y_{N^{(1)},2} & \cdots & y_{N^{(1)},N^{(2)}} & 0 & y^*_{N^{(1)},N^{(2)}} & y^*_{N^{(1)},N^{(2)}-1} & y^*_{N^{(1)},2} \end{bmatrix};$$

$\hat{\boldsymbol{S}} \leftarrow \mathrm{FT}^{(2)}\left(\hat{\boldsymbol{Y}}\right);$

$\mu \leftarrow \frac{1}{N^{(1)}(n_2 - n_1)} \sum_{n^{(1)}=1}^{N^{(1)}} \sum_{n^{(2)}=n_1}^{n_2} \hat{s}_{n^{(1)},n^{(2)}};$ *Mean of points in noise region*

$\sigma^2 \leftarrow \frac{1}{N^{(1)}(n_2 - n_1)} \sum_{n^{(1)}=1}^{N^{(1)}} \sum_{n^{(2)}=n_1}^{n_2} (\hat{s}_{n^{(1)},n^{(2)}} - \mu)^2;$ *Variance of points in noise region*

$\boldsymbol{W} \leftarrow$ RANDOMGAUSSIAN$(\mu, \sigma^2, N^{(1)}, 2N^{(2)});$

$c \leftarrow (r_0 + r_1)/2;$ *Center of super-Gaussian filter*

$b \leftarrow r_1 - r_0;$ *Width of super-Gaussian filter*

$\boldsymbol{g}^{(1)} \leftarrow \mathbf{1} \in \mathbb{R}^{N^{(1)}};$

$\boldsymbol{g}^{(2)} \leftarrow \mathbf{0} \in \mathbb{R}^{2N^{(2)}};$

for $n^{(2)} = 1, \cdots, 2N^{(2)}$ **do**

  $g^{(2)}_{n^{(2)}} \leftarrow \exp\left(-2^{p+1}\left(\frac{n^{(2)} - c}{b}\right)^p\right)$ *$p = 40$ by default*

end for;

$\boldsymbol{G} \leftarrow \boldsymbol{g}^{(1)} \otimes \boldsymbol{g}^{(2)};$

$\tilde{\boldsymbol{S}} \leftarrow \hat{\boldsymbol{S}} \odot \boldsymbol{G} + \boldsymbol{W} \odot (\mathbf{1} \in \mathbb{R}^{N^{(1)} \times 2N^{(2)}} - \boldsymbol{G});$ *See Figure S3*

$\lambda, \rho \leftarrow c - \left\lfloor \frac{\gamma b}{2} \right\rfloor, c + \left\lceil \frac{\gamma b}{2} \right\rceil;$

$$\bar{\boldsymbol{S}} \leftarrow \begin{bmatrix} \tilde{s}_{1,\lambda} & \tilde{s}_{1,\lambda+1} & \cdots & \tilde{s}_{1,\rho} \\ \tilde{s}_{2,\lambda} & \tilde{s}_{2,\lambda+1} & \cdots & \tilde{s}_{2,\rho} \\ \vdots & \vdots & \ddots & \vdots \\ \tilde{s}_{N^{(1)},\lambda} & \tilde{s}_{N^{(1)},\lambda+1} & \cdots & \tilde{s}_{N^{(1)},\rho} \end{bmatrix};$$

Continues on next page...

$\hat{\tilde{\boldsymbol{Y}}} \leftarrow \frac{\rho-\lambda}{N^{(2)}}\,\mathrm{IFT}^{(2)}\left(\bar{\boldsymbol{S}}\right)$;

$\epsilon \leftarrow \left\lfloor \frac{\rho-\lambda}{2} \right\rfloor$;

$$\tilde{\boldsymbol{Y}} \leftarrow \begin{bmatrix} \hat{\tilde{y}}_{1,1} & \hat{\tilde{y}}_{1,2} & \cdots & \hat{\tilde{y}}_{1,\epsilon} \\ \hat{\tilde{y}}_{2,1} & \hat{\tilde{y}}_{2,2} & \cdots & \hat{\tilde{y}}_{2,\epsilon} \\ \vdots & \vdots & \ddots & \vdots \\ \hat{\tilde{y}}_{N^{(1)},1} & \hat{\tilde{y}}_{N^{(1)},2} & \cdots & \hat{\tilde{y}}_{N^{(1)},\epsilon} \end{bmatrix};$$

return $\tilde{\boldsymbol{Y}}$

end procedure

Algorithm S6 Proposed simplified frequency-filtration procedure which wasn't used in this work (see the remark at the beginning of Section S1.4).

procedure FILTER($\boldsymbol{Y} \in \mathbb{C}^{N^{(1)} \times N^{(2)}}$, $\boldsymbol{r} \in [[1, 2N^{(2)}]]^2$,)

$$\hat{\boldsymbol{Y}} \leftarrow \begin{bmatrix} \mathrm{Re}(y_{1,1}) & y_{1,2} & \cdots & y_{1,N^{(2)}} & 0 & y^*_{1,N^{(2)}} & y^*_{1,N^{(2)}-1} & y^*_{1,2} \\ \vdots & \vdots & \vdots & \vdots & \vdots & \vdots & \vdots & \vdots \\ \mathrm{Re}(y_{N^{(1)},1}) & y_{N^{(1)},2} & \cdots & y_{N^{(1)},N^{(2)}} & 0 & y^*_{N^{(1)},N^{(2)}} & y^*_{N^{(1)},N^{(2)}-1} & y^*_{N^{(1)},2} \end{bmatrix};$$

$\hat{\boldsymbol{S}} \leftarrow \mathrm{FT}^{(2)}\left(\hat{\boldsymbol{Y}}\right)$;

$$\bar{\boldsymbol{S}} \leftarrow \begin{bmatrix} \tilde{s}_{1,r_1} & \tilde{s}_{1,r_1+1} & \cdots & \tilde{s}_{1,r_2} \\ \tilde{s}_{2,r_1} & \tilde{s}_{2,r_1+1} & \cdots & \tilde{s}_{2,r_2} \\ \vdots & \vdots & \ddots & \vdots \\ \tilde{s}_{N^{(1)},r_1} & \tilde{s}_{N^{(1)},r_1+1} & \cdots & \tilde{s}_{N^{(1)},r_2} \end{bmatrix};$$

$\hat{\tilde{\boldsymbol{Y}}} \leftarrow \frac{r_2-r_1}{N^{(2)}}\,\mathrm{IFT}^{(2)}\left(\bar{\boldsymbol{S}}\right)$;

$\epsilon \leftarrow \left\lfloor \frac{r_2-r_1}{2} \right\rfloor$;

$$\tilde{\boldsymbol{Y}} \leftarrow \begin{bmatrix} \hat{\tilde{y}}_{1,1} & \hat{\tilde{y}}_{1,2} & \cdots & \hat{\tilde{y}}_{1,\epsilon} \\ \hat{\tilde{y}}_{2,1} & \hat{\tilde{y}}_{2,2} & \cdots & \hat{\tilde{y}}_{2,\epsilon} \\ \vdots & \vdots & \ddots & \vdots \\ \hat{\tilde{y}}_{N^{(1)},1} & \hat{\tilde{y}}_{N^{(1)},2} & \cdots & \hat{\tilde{y}}_{N^{(1)},\epsilon} \end{bmatrix};$$

return $\tilde{\boldsymbol{Y}}$

end procedure

Algorithm S7 Procedure for grouping estimated signals which belong to the same multiplet structure. HashMap$\langle K, V\rangle$() generates an empty hashmap, which will comprise key-value pairs of types K and V, respectively. Array$\langle V\rangle$() generates an empty array, which will comprise entries of type V. Append(A, x) increases the size of array A by 1 and adds x to the end of it. Add(H, K, V) adds the key-value pair (K, V) to hashmap H. Remove(H, K) removes the key-value pair with key K from the hashmap H.

```
procedure AssignMultiplets( θ^(*) ∈ R^{6M}, ϵ ∈ R_{>0} )
    H ← HashMap⟨N, Array⟨N⟩⟩();
    for m = 1, ···, M do
        b ← False;                                  ▷ Flag indicating whether the signal falls into an existing group
        f_m^(1), f_m^(2) ← θ^(*)_{2M+m}, θ^(*)_{3M+m};
        f_c ← f_m^(2) − f_m^(1);
        for f'_c, A in H do
            if |f_c − f'_c| < ϵ then;
                b ← True;
                f_old ← f'_c;
                S ← Size(A);
                f_new ← (S f_old + f_c)/(S + 1);     ▷ Compute the new mean frequency of the group
                Append(A, m);
                Remove(H, f_old);                    ▷ Replace the new frequency with the old one
                Add(H, f_new, A);
                break;
            end if
        end for
        if not b then
            A ← Array⟨N⟩();
            Append(A, m);
            Add(H, f_c, A);
        end if
    end for
    return H;
end procedure
```

S7 Code Listings

Code Listing S1: 2DJ pulse sequence for use in SPINACH.

```
function fid = jres_seq(spin_system, parameters, H, R, K)
    % Compose Liouvillian
    L = H + 1i * R + 1i * K; clear('H', 'R', 'K');
    % $\tau^{(1)}$
    d1 = 1 / parameters.sweep(1);
    % $\tau^{(2)}$
    d2 = 2 / parameters.sweep(2)
    % $N^{(1)}$
    N1 = parameters.npoints(1);
    % $N^{(2)}$
    N2 = parameters.npoints(2);
    % Targeted nucleus (always $^{1}$H)
    nuc = parameters.spins{1}
    % Initial state
    rho = state(spin_system, 'Lz', nuc);
    % Detection state
    coil = state(spin_system, 'L+', nuc);
    % Get the pulse operators
    Lp = operator(spin_system, 'L+', nuc);
    Lx = (Lp + Lp') / 2;
    % First pulse: 90° about x
    rho = step(spin_system, Lx, rho, pi / 2);
    % First half of t1 evolution
    rho = evolution(spin_system, L, [], rho, d1 / 2, N1 - 1, 'trajectory');
    % Select "-1" coherence
    rho = coherence(spin_system, rho, {{nuc, -1}});
    % Second pulse: 180° about x
    rho = step(spin_system, Lx, rho, pi);
    % Select "+1" coherence
    rho = coherence(spin_system, rho, {{nuc, +1}});
    % Second half of t1 evolution
    rho = evolution(spin_system, L, [], rho, d1 / 2, N1 - 1, 'refocus');
    % Run the F2 evolution
    fid = evolution(spin_system, L, coil, rho, d2, N2 - 1, 'observable');
end
```

Code Listing S2: Python function used to transform a PSYCHE interferogram into a 1D FID.

```python
from nmrespy.sig import convdta
import numpy as np

def process_psyche(ser: np.ndarray, grpdly: float, sw1: float, sw2: float) -> np.ndarray:
    """Process a raw PSYCHE ser array to form a 1D pure shift FID.

    Parameters
    ----------

    ser
        2D PSYCHE interferogram, i.e. the contents of a Bruker ser file.

    grpdly
        Group delay.

    sw1
        Sweep width in the indirect dimension (Hz).

    sw2
        Sweep width the direct dimension (Hz).
    """
    drop_pts = 4
    chunk_pts = sw2 // sw1
    # `convdta` mimics Bruker's function of the same name, which performs
    # digital to analogue conversion
    fid = convdta(ser, grpdly)
    # Slice to retain the initial points of each direct-dimension signal.
    fid = fid[:, drop_pts : drop_pts + chunk_pts]
    # Flatten into a 1D FID
    fid = fid.flatten()
    return fid
```

References

[1] S. G. Hulse. “Estimation of NMR signals in the time domain: methodology, applications and software”. PhD thesis. University of Oxford, 2023. DOI: 10.5287/ora-bpdg8rr9k.

[2] S. G. Hulse and M. Foroozandeh. “Newton meets Ockham: parameter estimation and model selection of NMR data with NMR-EsPy”. In: *Journal of Magnetic Resonance* 338 (2022), p. 107173. DOI: 10.1016/j.jmr.2022.107173.

[3] J. Nocedal and S. J. Wright. *Numerical optimization*. 2nd ed. Springer series in operations research. New York: Springer, 2006. DOI: 10.1007/978-0-387-40065-5.

[4] N. I. Fisher. *Statistical Analysis of Circular Data*. Cambridge: Cambridge University Press, 1993. ISBN: 9780511564345. DOI: 10.1017/cbo9780511564345.

[5] Y. Pawitan. *In All Likelihood: Statistical Modelling and Inference Using Likelihood*. Oxford: Oxford University Press, 2001. ISBN: 9780199671229.

[6] Y. Hua and T. K. Sarkar. “Matrix pencil method for estimating parameters of exponentially damped/undamped sinusoids in noise”. In: *IEEE Trans. on Acoust. Speech Signal Process.* 38.5 (1990), pp. 814–824. DOI: 10.1109/29.56027.

[7] Y. Hua. “Estimating two-dimensional frequencies by matrix enhancement and matrix pencil”. In: *IEEE transactions on signal processing* 40.9 (1992), pp. 2267–2280. DOI: 10.1109/78.157226.

[8] F. - J. Chen, C. C. Fung, C. - W. Kok and S. Kwong. “Estimation of two-dimensional frequencies using modified matrix pencil method”. In: *IEEE transactions on signal processing* 55.2 (2007), pp. 718–724. DOI: 10.1109/tsp.2006.885813.

[9] W. E. Arnoldi. “The principle of minimized iterations in the solution of the matrix eigenvalue problem”. In: *Quarterly of Applied Mathematics* 9.1 (1951), pp. 17–29. DOI: 10.1090/qam/42792.

[10] `scipy.sparse.linalg.svds`. https://docs.scipy.org/doc/scipy/reference/generated/scipy.sparse.linalg.svds.html. Accessed 15-08-2023.

[11] J. Rissanen. “Modeling by shortest data description”. In: *Automatica* 14.5 (1978), pp. 465–471. DOI: 10.1016/0005-1098(78)90005-5.

[12] M. Wax and T. Kailath. “Detection of signals by information theoretic criteria”. In: *IEEE Trans. Acoust. Speech Signal Process.* 33.2 (1985), pp. 387–392. DOI: 10.1109/tassp.1985.1164557.

[13] Y. - Y. Lin, P. Hodgkinson, M. Ernst and A. Pines. “A novel detection–estimation scheme for noisy NMR signals: applications to delayed acquisition data”. In: *Journal of Magnetic Resonance* 128.1 (1997), pp. 30–41. DOI: 10.1006/jmre.1997.1215.

[14] M. H. Levitt. *Spin dynamics: basics of nuclear magnetic resonance.* 2nd edition. Chichester: John Wiley & Sons, Ltd, 2008. ISBN: 9780470517123.

[15] M. Mayzel, K. Kazimierczuk and V. Y. Orekhov. "The causality principle in the reconstruction of sparse NMR spectra". In: *Chem. Commun.* 50.64 (2014), pp. 8947–8950. DOI: 10.1039/c4cc03047h.

[16] D. Gołowicz, P. Kasprzak and K. Kazimierczuk. "Enhancing compression level for more efficient compressed sensing and other lessons from NMR spectroscopy". In: *Sensors (Basel)* 20.5 (2020), p. 1325. DOI: 10.3390/s20051325.

[17] G. Bodenhausen, R. Freeman and D. L. Turner. "Suppression of artifacts in two-dimensional J spectroscopy". In: *Journal of Magnetic Resonance (1969)* 27.3 (1977), pp. 511–514. DOI: 10.1016/0022-2364(77)90016-6.

[18] M. Foroozandeh, R. W. Adams, P. Kiraly, M. Nilsson and G. A. Morris. "Measuring couplings in crowded NMR spectra: Pure shift NMR with multiplet analysis". In: *Chemical Communications* 51.84 (2015), pp. 15410–15413. DOI: 10.1039/c5cc06293d.

[19] H. J. Hogben, M. Krzystyniak, G. T. P. Charnock, P. J. Hore and I. Kuprov. "Spinach – A software library for simulation of spin dynamics in large spin systems". In: *Journal of magnetic resonance (1997)* 208.2 (2011), pp. 179–194. DOI: 10.1016/j.jmr.2010.11.008.
